# Bathymetry of the Antarctic continental shelf and ice shelf cavities from circumpolar gravity anomalies and other data

**DOI:** 10.1038/s41598-024-81599-1

**Published:** 2025-01-07

**Authors:** Raphaelle Charrassin, Romain Millan, Eric Rignot, Mirko Scheinert

**Affiliations:** 1 Department Earth System Science, University of California Irvine, Irvine, CA 92697 USA; 2 Université Grenoble Alpes, CNRS, IRD, INP, 38400 Grenoble, Isère France; 3 Jet Propulsion Laboratory, California Institute of Technology, Pasadena, CA 91107 USA; 4 Department Civil and Environmental Engineering, University of California Irvine, Irvine, CA 92697 USA; 5 TUD Dresden University of Technology, Chair of Geodetic Earth System Research, 01062 Dresden, Germany

**Keywords:** Physical oceanography, Cryospheric science

## Abstract

Bathymetry critically influences the intrusion of warm Circumpolar Deep Water onto the continental shelf and under ice shelf cavities in Antarctica, thereby forcing ice melting, grounding line retreat, and sea level rise. We present a novel and comprehensive bathymetry of Antarctica that includes all ice shelf cavities and previously unmeasured continental shelf areas. The new bathymetry is based on a 3D inversion of a circumpolar compilation of gravity anomalies constrained by measurements from the International Bathymetric Chart of the Southern Ocean, BedMachine Antarctica, and discrete seafloor measurements from seismic and ocean robotic probes. Previously unknown troughs with thicker ice shelf cavities are revealed in many parts of Antarctica, especially East Antarctica. The greater depths of troughs on the continental shelf and ice shelf cavities imply that many glaciers are more vulnerable to ocean subsurface warming than previously thought, which may increase the projections of sea level rise from Antarctica.

**Supplementary Information:**

The online version contains supplementary material available at 10.1038/s41598-024-81599-1.

## Introduction

In Antarctica, glaciers are buttressed by ice rises and floating extensions of the ice sheet named “ice shelves”, which regulate the discharge of ice from the continent in the Southern Ocean^1^. As more subsurface, warm and saline ocean water of Circumpolar Deep Water (mCDW) origin is pushed toward Antarctica by prevailing westerly winds, the ice shelves basal melt increased, eroding more quickly ice shelves into the ocean. This loss of ice mass reduces the ability of ice shelves to retain glacier flow, which increases the contribution of Antarctica to sea level rise^2,3^. To project the evolution of Antarctica, it is essential to understand where and how warm CDW intrudes on the continental shelf, flows down seafloor pathways into ice shelf cavities, and reaches the ice shelf grounding zone to melt basal ice^4,5^. In places where the bathymetry is shallow (<300 m), CDW may be blocked from accessing the glaciers. In this case, the ice shelf thickness may also play a role in affecting the cavity shape. Conversely, if the seafloor topography is deep (650–750 m, e.g. around Pine island glacier^5^), CDW will easily access ice shelf cavities. While extensive bathymetry mapping has been conducted in a number of critical sectors, there are many parts of Antarctica where bathymetry is incomplete or non-existent^6^, which limits our ability to understand ongoing changes and project future changes.

Bathymetry is primarily measured from icebreaking ships equipped with acoustic multibeam or single beam echo sounders (MBES, SBES)^6^. The presence of extensive sea ice cover and stranded icebergs in front of the ice shelves makes the navigation difficult. Consequently, vast territories are devoid of seafloor soundings in Antarctica. Mapping bathymetry beneath ice shelves presents additional challenges requiring seismic surveying^7^, autonomous underwater vehicles (AUV)^8–11^, or Conductivity, Temperature, Depth (CTD) data collected through bore holes in the ice, which is done at discrete locations. Although many ice shelves have been subject to these techniques at extensive scales, adequate coverage is lacking on all but eight of the 160 largest ice shelves (PIG, Ronne, Ross, Fimbul, Larsen C, Amery, Totten, George VI), due to the challenges of implementing them^6,12^.

Free-air gravity anomalies (which are technically disturbances, but will be referred as free-air anomalies for conventions^13^) inform about variations in water thickness and bedrock-sediment density beneath ice shelves. Prior studies have shown that a three-dimensional (3D) inversion of gravity data, constrained by observations of seafloor depth, is an effective, physics-based method for modelling bathymetry^7,12,14–17^. In-situ constraints include MBES and SBES data, seismic recordings, and depth recordings from CTD. The nominal precision of a gravity inversion is ±60 m with gravity data collected with a precision at the milligal level^15,18^. Gravity data is also wavelength limited based on depth to source and filtering applied in post processing^14,16,19–21^. A key feature of recent inversions is to offer a smooth transition with models of bed topography beneath the grounded ice, e.g. BedMachine Antarctica v3.7 (BMv3.7)^22^, and with the International Bathymetric Chart of the Southern Ocean (IBCSOv2)^6^ to avoid discontinuities at domain boundaries.

Here, we employ a novel, comprehensive, compilation of gravity anomalies, named AntGG2021, which superseeds a prior version, named AntGG2016^23^ to infer the bathymetry of the continental shelf and ice shelf cavities. We present the data and methods, observational constraints, inversion results, and performance assessment. We discuss key regions of Antarctica and how the new bathymetry will improve our understanding of ice-ocean interaction and glacier evolution.

## Methods

### AntGG2021 gravity compilation

Ground-based and airborne gravity measurements have been compiled in the frame of the International Association of Geodesy Sub-Commission 2.4f “Gravity and Geoid in Antarctica” (AntGG)^23^. A first compilation of combined and gridded gravity anomalies was published by^23^, hereafter called AntGG2016. This compilation covered about 73% of the Antarctic continent and had a grid resolution of 10 km. Since then, a number of new gravity data sets have been made available, especially over regions where in-situ data were sparse or missing, e.g. at South Pole^24^, in Dronning Maud Land, East Antarctica^25^, over the Pensacola Basin^26^ or in Marie Byrd Land, West Antarctica^27^. Applying an improved processing workflow and taking all available in-situ gravity data into account led to a new compilation^28^, AntGG2021.The grid has a resolution refined from 10 to 5 km, and covers all of Antarctica south of $60^{\circ }$S, providing different quantities. In this study, we use the AntGG2021 gravity anomaly product created by Scheinert et al.^29^ and available at PANGEA (https://doi.pangaea.de/10.1594/PANGAEA.971238).

The improved processing scheme incorporates the remove-compute-restore (RCR) technique^30^. This method in geodesy helps align different gravity data sets with an a-priori Earth gravity model based on satellite and topographic data^31^. During this procedure, long-wavelengths as well as short-wavelengths signal parts are removed from the original gravity observations. We use a high-resolution gravity model SATOP-1^31^ to compute the reduction (residual gravity anomalies) directly at the respective measurement location. The SATOP-1 model is inferred in the spheroidal-harmonic domain from a weighted combination of a satellite-only model and a high-resolution topographic model EARTH2014^32,33^ (see Supplementary Information).

For the computation step, a partition-enhanced least-squares collocation (LSC) method is applied^34^ which incorporates the stochastic properties of the residual gravity data and allows to compute the desired gravity anomalies at any given point in space, thus, also on a regular grid with the desired 5 km resolution as well as at different height levels, which implicitly contains the downward continuation to the ice surface elevation. It is an advantage of LSC that all desired quantities are computed as functionals of the disturbing potential, $T$, and that the method provides an accuracy measure (see Supplementary Information). This provides a unified theoretical framework, and ensures that all computed quantities are consistent with each other. Finally, the contribution of the background model is restored directly at the grid points.

**Fig. 1:**
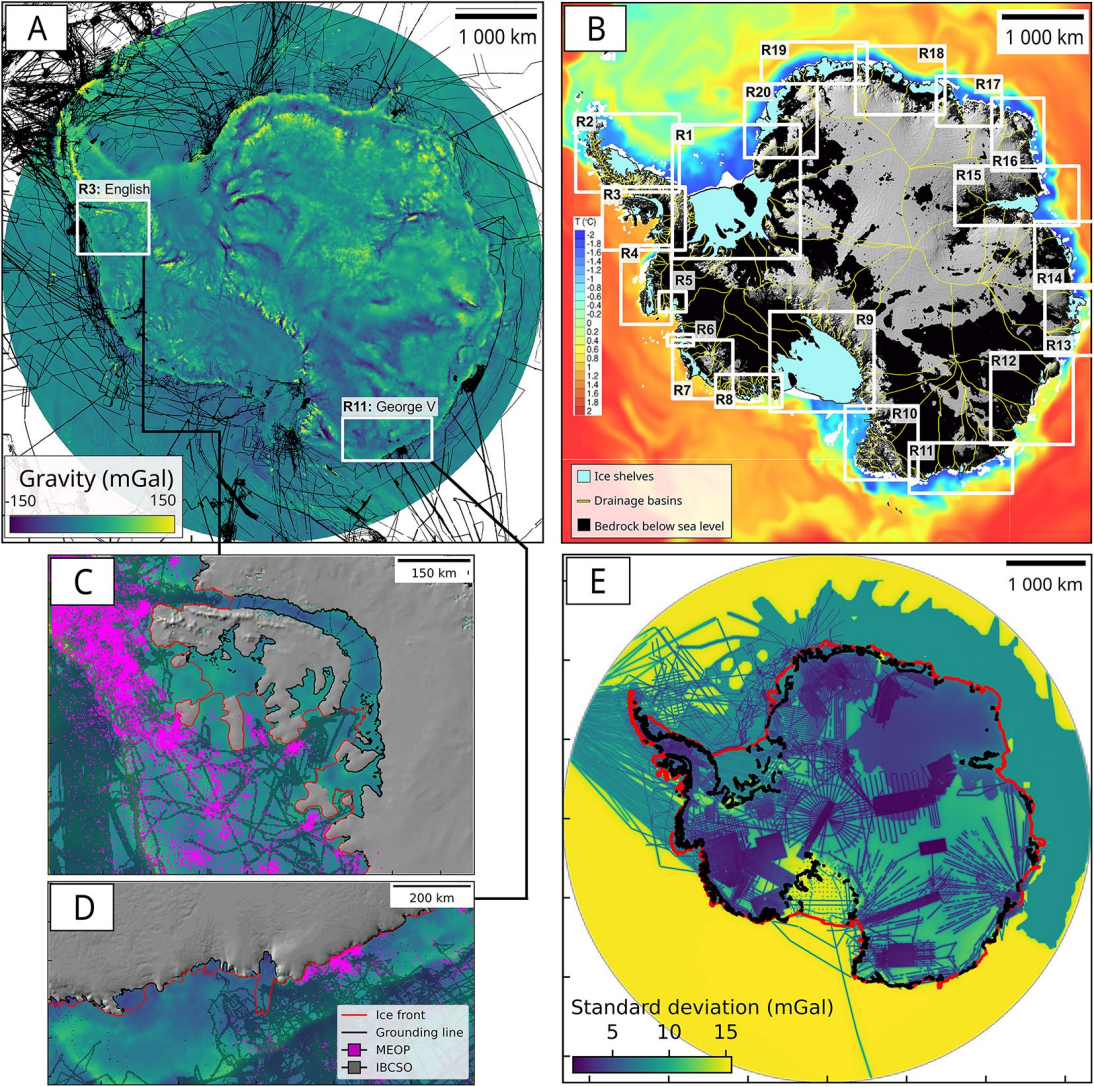
Gravity data and constraints for the inversion. (**A**) AntGG2021 free-air gravity anomalies color coded from −150 mGal (1 Gal = 1 m/$\hbox {s}^{2}$) to +150 mGal with location of MBES/SBES from^6^. (**B**) Geographic names and ocean temperature at 310-m depth from the Southern Ocean State Estimate (SOSE) model^61^ color-coded from cold ($-2\,^{\circ }$C, blue) to warm ($+2\,^{\circ }$C, red). White areas are less than 310 m depth. Ice shelves are light blue. Bed beneath grounded ice is black if below sea level, grey otherwise. Zoom in on (**C**) English and (**D**) George V Coasts with bathymetry from MEOP^41^ in pink and IBCSOv2 or seismic in black. (**E**) Standard deviation map of free-air gravity anomalies from AntGG2021. The map is color coded from 1.5 mGal (blue) to +15.5 mGal (yellow). Standard deviations of 15 mGal indicates regions that are not covered by terrestrial gravity measurements.

As we move away from ground or airborne data, the gravity grid of AntGG2021 becomes closer to the background gravity model SATOP-1^31^, at a lower resolution, hence resulting in higher uncertainties in gravity (Fig. S1). In regions with no terrestrial data, the larger errors of SATOP-1 are expected to be outweighted due to the regularized least-squares adjustment strategy when combining the satellite with the topographic data (see above). In these areas with no terrestrial observations, standard deviations values are exceeding 15 mGal (Fig. 1E). Regions covered by terrestrial gravimetry data have standard deviations typically ranging between 2 and 8 mGal.

### Model setup

For each ice shelf region (Fig. S2), the model domain includes an ice layer (density 0.9167 g/$\hbox {cm}^{3}$), a water layer (density 1.028 g/$\hbox {cm}^{3}$), and a bedrock layer (density of 2.67 g/$\hbox {cm}^{3}$). For the bedrock elevation beneath grounded ice, we use BMv3.7, which has been fed with IBCSOv2^6^. For ground observation seafloor depths, we use MBES and SBES data in IBCSOv2. The method of^16^ is used to account for changes in bedrock density (see section “Gravity inversion”).

Seismic data is available for Pine Island, Larsen C, Fimbul, Amery, George VI, Ross, Ronne and Totten ice shelves^35–40^ (see Table S2). We use AUV data for Pine Island^8^, CTD data from the Hadley Centre (bodc.ac.uk) between 1960 and 2019, and seafloor depths from the Marine mammals Exploring the Ocean Pole to Pole project (MEOP)^41^. For each available mammal dive, we calculate a maximum dive depth and assemble an Antarctic-wide maximum dive depth map (Fig. 1C,D; Fig. S3). We calculate the maximum dive depth, median, and standard deviation inside a rolling window of 10x10 km. If the 2-$\sigma$ value is greater than 100-m (i.e., the expected accuracy of the gravity inversion^18,19^), we use the dive with the maximum depth. Note that max dive depth indicates regions where the bathymetry could potentially be deeper, but not shallower. In other cases, we use the average dive depth within the 10x10 km window, and merged the final layer into BMv3.7 using bilinear interpolation.

### Gravity inversion

We used the approach of Parker’s 1973^42^, which applies a series of Fourier transforms to calculate the gravitational anomaly generated by a layer of material with uneven density, inside the Geosoft GM-SYS 3D software. A forward model of the gravity anomalies associated with an initial bedrock elevation (compilation of IBCSOv2, MEOP, seismic and AUV) is then calculated. The forward gravity signal is compared to AntGG2021 and the bedrock is iteratively migrated until the misfit between modeled and observed gravity anomaly is less than a arbitrary set threshold of 0.1 mGal^15,18^. Experiments with thresholds ranging from 0.1 to 5 mGals revealed no significant changes in bedrock topography, and no overfitting, suggesting that the first iteration provides the largest changes in seafloor elevation. During the inversion procedure, only the bedrock topography is authorized to move downward or upward, meaning that a new water column thickness is calculated at each iteration. The new model geometry is then re-forward calculated. To account for spatial variations in the long-wavelength regional component of the gravity signal due to bedrock geology, we follow the approach in^16^ (“DC-shift” approach), which uses the misfit between observed and modeled gravity at locations of known seafloor depths and interpolates the signal in between using a minimum curvature algorithm. The interpolated grid is then added to the original gravity data. This method has shown comparable performances with density inversion methods, with the advantage of lower computational time since only one inversion is needed. This is of particular interest when conducting large scale inversion all around the Antarctic coast (see Fig. 1).

We conduct the inversion around the entire continent, including all ice shelf cavities, divided into 20 subregions (R1–R20) to minimize the computational cost (Fig. S1). In the final inversion product, the bathymetry conserves pre-existing seafloor depth measurements, which were also used as constraints during the inversion procedure. As the inversion might be really sensitive to local isolated data, and to high-frequency free-air gravity anomalies^16,43^, strong changes in the gravity signal or outliers may alter the precision of the inversion. We follow a multi-step filtering procedure. From an initial inversion, we identify poor performance areas based on abrupt transitions or low water column thickness ($<30$ m). Indeed, a very thin water column indicates a region where the ice could be potentially grounded. If this has never been detected by SAR interferometry mapping^44^, we posits that our inversion is failing at recovering the seafloor depth. We smooth out high-frequency variations in the misfit gravity grid using a low-pass filter (between 7.5 and 15 km width) and re-run the inversion. If poorly performing regions are still present, we mask the bathymetric misfits and use a minimum curvature interpolation scheme to fill the gap (Fig. S3).

### Inversion error assessment

We quantify the error related to the inversion process, by calculating the misfit between observed and modeled gravity, which we translate in meters using a conversion factor of 5.8 mGal per hundred meters^14,15^.The misfit value provides an estimate of how well our method can fit the observed gravity signal. The performance of the inversion is also quantified by comparing the inversions with seismic measurements when available. To accurately quantify the uncertainty on our method, we perform three inversions test in rectangular regions of 200 km by 125 km, with dense MBES coverage, and where the standard deviation of AntGG varies from a minimum value of 4.9 mGal to a maximum value of 8.1 mGal. The tests are located in front of the Brunt–Stancomb ice shelf (Fig. S4), the Jelbart ice shelf (Fig. S5) (rectangular region of 250 km by 185 km) and the George VI ice shelf (Fig. S6), respectively centered at $67.242^{\circ }$W, $38.097^{\circ }$N; $68.773^{\circ }$W, $4.586^{\circ }$S and $73.473^{\circ }$W, $27.590^{\circ }$S. The three domains have a extensive free-air gravity anomalies coverage, with a mean standard deviation as follows, 5.58 ± 2.02 mGal (Fig. S4-H); 7.08 ± 1.94 mGal (Fig. S5-H) and 7.8 ± 0.96 mGal (Fig. S6-H) respectively. The inversion domain offshore of Jelbart ice shelf is the only one located on the abyssal plain. We only use MBES data along the periphery to constraint the inversion, and remove available MBES in the center. The accuracy is finally quantified by comparing the results with the actual “unseen” MBES data^16^. The actual accuracy of the inversion beneath ice shelves will be critically influenced by the coverage and resolution of the available airborne gravity data and distribution of observed tie points. However, a part of the quality of the fit between recovered bathymetry and unseen MBES data (e.g. Fig. S4-G), especially at high frequencies, can also be explained by the inclusion of topographic information in the inference of the AntGG2021 model (via the a-priori SATOP-1 model), which can potentially “leak” into the final inversion results.

## Results

**Fig. 2:**
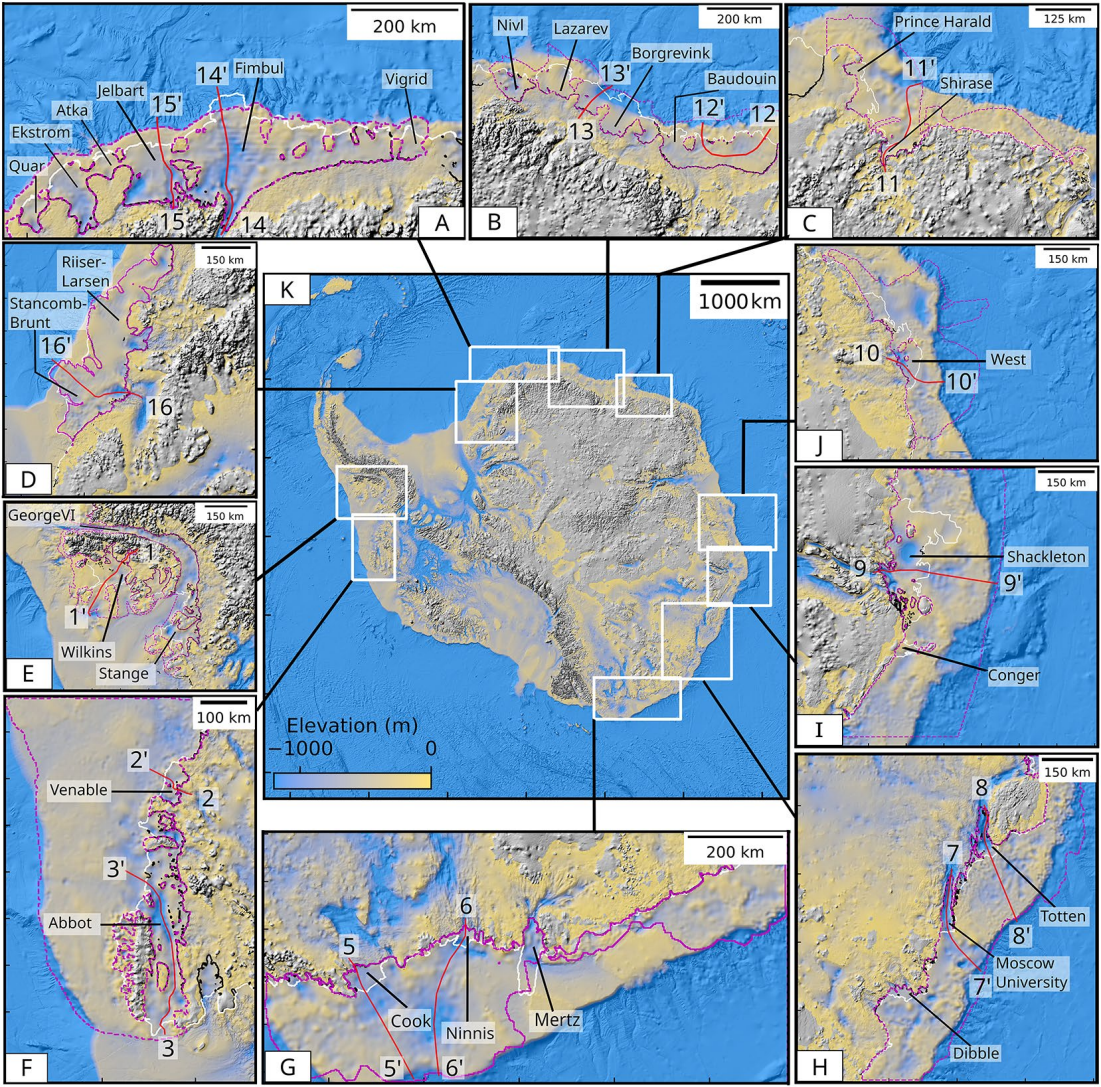
Bathymetry of Antarctica color coded from −1250 m (blue) to 0 m (yellow) with shaded relief for 10 regions: (**A**) Jebart and Fimbul (R19), (**B**) Borgrevink and Baudouin (R18), (**C**) Shirase (R17), (**D**) Brunt–Stancomb Wills (R20), (**E**) George VI (R3), (**F**) Abbot and Venable (R4), (**G**) Cook, Ninnis, Mertz (R11), (**H**) Moscow, Totten (R12), (**I**) Shackleton (R13) and (**J**) West (R14), with ice shelf boundary in white and inversion domains in purple. Profiles shown in Fig. 3 are red. (**K**) Overview of the 10 sub-regions.

Our product is an Antarctic-wide gravity inversion for bathymetry (referred to the geoid), including all ice shelves. We obtain an calculated gravity inversion misfit of −0.1 ± 1.6 mGal, which translates into a nominal inversion elevation misfit of −1.5 ± 27.5 m. This quantity represents the remainder of the gravity signal that the inversion is not able to fit. The performance of the inversion is also quantified for each domain, by calculating statistic of the differences between the bathymetry and available seismic measurements (mean ± standard deviation, see below). Inside the test boxes, the comparison between inverted bathymetry and unseen data shows differences of −15 ± 105 m (George VI), 2 ± 123 m (Brunt–Stancomb) and 23 ± 176.3 m (Jelbart) (Fig. S6-G; S4-G; S5-G). For higher precision gravity measurements conducted in Greenland (1.5 mGal at crossovers), differences with MBES of −10 ± 60 m were reported^16^. These different experiments allows us to provide an estimate of the inversion uncertainty as a function of the gravity standard deviation using a linear fit between the standard deviation from the AntGG2021 (Fig. 1C) and the standard deviation of the difference between inversion results and unseen bathymetry data. The final uncertainty, is about 162 ± 46 m (mean±standard deviation), over all our inversion domains (see Supplementary Table 3  ; Fig. S9). It is important to note that uncertainties may be underestimated, and could reach several hundreds of meters, in regions with no terrestrial gravity measurements. These are the places where lack of airborne gravity data is critical (e.g. Fig. 2G,H).

For regions lacking in-situ data, uncertainties in bed density introduce larger errors that are difficult to quantify^16^. Uncertainties in free air gravity anomaly (Fig. S1) are the highest between Victoria Land and Queen Mary coast, which are devoid of airborne or ground data and therefore mostly rely on SATOP-1.

Overall, our new product shows deeper bathymetry in the majority of the regions with an overall difference with BedMachine v3 of 56 ± 160 m (mean ± standard deviation). However, we observe that local differences, at the scale of channels, can be largely higher and reach several hundreds of meters, which has the potential to directly impact CDW penetration on the continental shelf and the glacier’s grounding lines. We both found large changes in bathymetry in West Antarctica (e.g. Wilkins, Venable, Abbott, etc.) and East Antarctica (e.g. West, Moscow, Denman, Shirase, Borgrevink and Jelbart, etc.). In the following section we describe these differences in detail and compare them with earlier products.

**Fig. 3:**
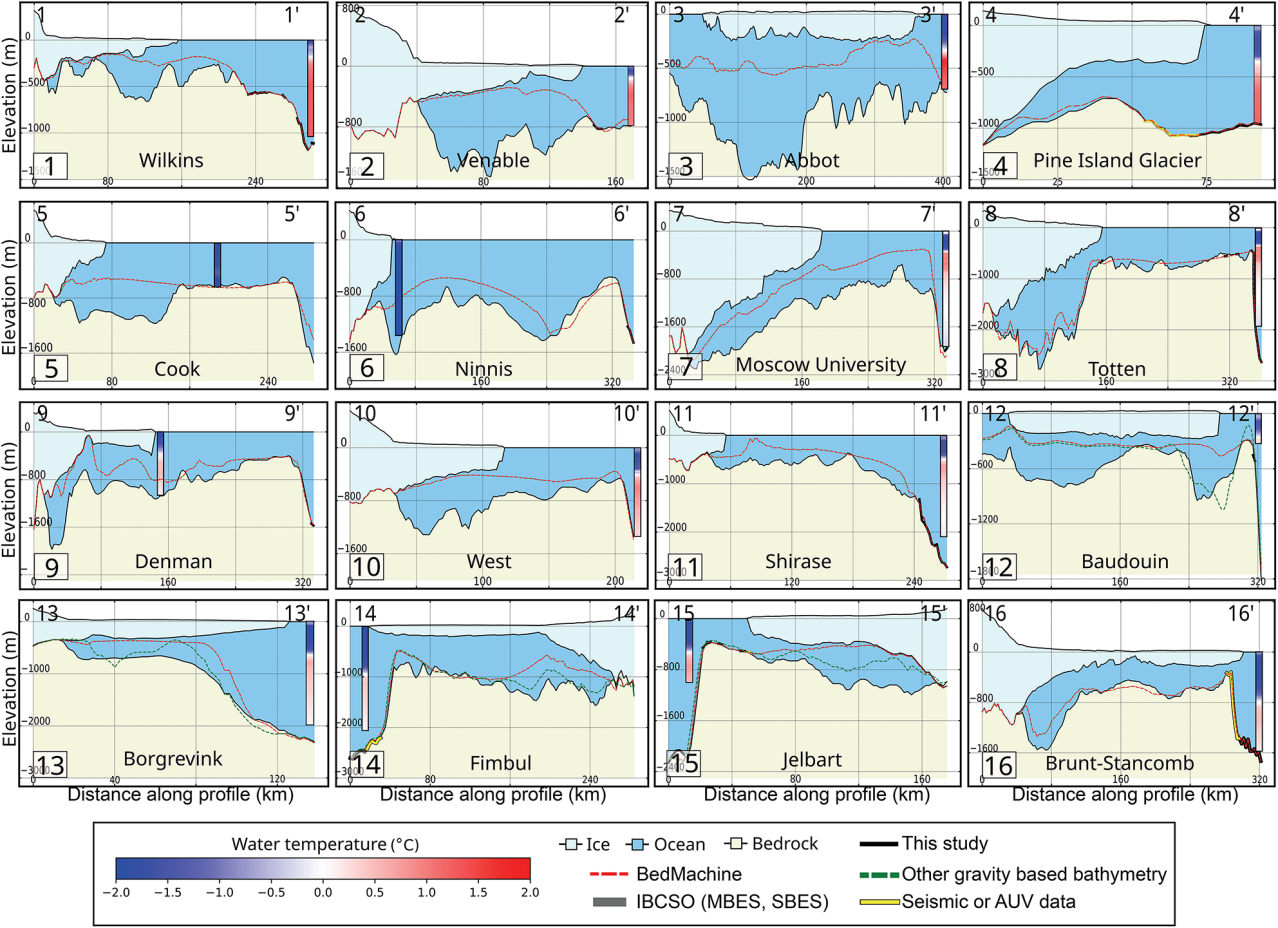
Comparison of AntGG2021 bathymetry with other data sources along specific profiles shown in Fig. 2 for 12 regions, and Pine Island Ice Shelf in Fig. S7e. Ice is light blue, ocean is blue, bedrock is light brown, IBCSOv2 is dotted red, new bathymetry is black, and observations are thick black. CTD observations are color coded from $-2\,^{\circ }$C to $2\,^{\circ }$C, with geographic locations in Fig. S8.

In the Antarctic Peninsula (R2–R3), the bathymetry of Larsen C (46,465 $\hbox {km}^{2}$ in size) and D (22,548 $\hbox {km}^{2}$) reveals a deeper bed with previously unknown channels on the continental shelf. Subglacial valleys up to 1000 m deep and 200–400 m deeper than IBCSOv2 are found in Whirlwind and Mobil Oil Inlets (Fig. S7b). On Larsen D, the bathymetry is up to 600 m deeper than IBCSOv2 with an east-west trough at 1200 m depth along Wilkins coast. The new bathymetry is 98 ± 176 m deeper on average. We fit seismic data^45^ within 2 ± 44 m. Beneath Wilkins Ice Shelf in R3, the bathymetry is up to 600 m deeper than IBCSOv2 and 650 m deeper beneath Stange Ice Shelf (Fig. 3). For George VI Ice Shelf (23,434 $\hbox {km}^{2}$), the bathymetry is 57 ± 133 m deeper than prior inversions^46^ (Table S1). We fit seismic data within 4 ± 43 m^47^ (Fig. S7c).

In West Antarctica (R4–R9), Abbot (29,688 $\hbox {km}^{2}$) and Venable (3194 $\hbox {km}^{2}$) have bathymetry considerably deeper (>400 m) than IBCSOv2 (Fig. S7d). We find a 100-km wide trough, 1500 m in depth, along the middle of Abbot 400 m deeper in IBCSOv2. The trough extends across the continental shelf. The bathymetry in R4 is 77±173 m deeper on average (Table S1). Our inversion is an improvement to the^21^ inversion. Indeed, this study uses more radar sounding data, that were measured by Operation Icebridge between 2014 and 2018. We also have larger MBES coverage, which increased south of $60 ^{o}$ S from 15.4% in IBCSOv1 to 25% in IBCSOv2^6,48^. We also use 3D inversions, unlike the 2D inversions in^21^, which allows to better account for changes in bedrock geology variations (through density inversion or DCShift method here). Indeed, two dimensions inversions can only account for geological changes along individual flight lines. In contrast, our 3D approach enables us to account for geological variations laterally, across all lines simultaneously and over the inversion domain.

The bathymetry beneath Cosgrove and Pine Island (R5) is revised from^14^ (Fig. 3). The seafloor is 47 ± 113 m deeper than IBCSOv2 (Table S1). Cosgrove is 150–300 m deeper near the grounding line and 950 m deep at the ice front. Pine Island is 100 m deeper at the grounding line as ambiguities in ice thickness are resolved. We fit the seismic and AUV data within 1 ± 38 m (Fig. S7e). Getz Ice Shelf (34,018 $\hbox {km}^{2}$) in R7 is similar to^43^ (Fig. S7g). The bathymetry is 16 ± 98 m deeper, with no change in sill depth between east and west Getz. In R8, Hull, Land, and Nickerson ice shelves (6500 $\hbox {km}^{2}$) have deeper bathymetry, with more pronounced troughs than in IBCSOv2, especially along Balchen Glacier. Along Ruppert Coast and Sulzberger Ice Shelf (12,333 $\hbox {km}^{2}$), the bathymetry is similar to IBCSOv2, with a succession of parallel channels up to 1500 m deep extending on the continental shelf (Fig. S7h). The bathymetry is 98 ± 196 m deeper on average (Table S1).

Ross Ice Shelf (500,809 $\hbox {km}^{2}$) (R9) benefits from a dense network of seismic data. The seafloor depth is 2300 m at the mouth of Byrd Glacier, rises at 550 m following the ice flow direction and goes down to 1900 m. This step in bathymetry is probably due to an artefact in AntGG2021 that was manually removed, but still has a partial impact on the result. East-west oriented artefacts in the gravity grid are also present below Ross. On average, the new bathymetry is 25 ± 101 m deeper (Table S1). We fit seismic data within 0 ± 101 m (Fig. S7i). Data from the ROSETTA-ice are currently missing from the new AntGG compilation and should be added in a future version.

In East Antarctica (R10–R20), R11 along George V Coast, the ANTGG compilation mostly rely on the background model. Hence uncertainties are difficult to quantify and could reach several hundreds of meters. Our inversion reveals new large scale bathymetric depressions on the continental shelf in front of Cook (3462 $\hbox {km}^{2}$), Ninnis (1899 $\hbox {km}^{2}$) and Mertz (5522 $\hbox {km}^{2}$) at 1200 m, 1600 m and 1300 m depth, respectively (Fig. 2, Fig. S7k) that extend on the continental shelf. The new bathymetry is 500, 300, and 600 m deeper beneath Cook, Mertz, and Ninnis, respectively, and 73 ± 170 m deeper on average (Table S1). This region is one of the most uncertain of all inversion domain, and errors could reach several hundreds of meters.

Holmes (1921 $\hbox {km}^{2}$), Moscow University (5798 $\hbox {km}^{2}$), Totten (6032 $\hbox {km}^{2}$) and Vincennes Bay in R12 are improved compared to prior work^49^ due to the 3D inversion, new seismic^40^, and MBES/SBES data^50^. The bathymetry is 73 ± 230 m deeper in average (Table S1), up to 600 m beneath Moscow. Large scale depression are also visible extending from the ice front further away on the continental shelf (Fig. 2 and 3). We fit seismic data within 37 ± 81 m (Fig. S7l).

For Shackleton (26,080 $\hbox {km}^{2}$) (R13), we find a broader trough beneath the ice shelf, extending across the continental shelf, 1000 m deeper than IBCSOv2 (Fig. S7m, Fig. 3). On average, the bathymetry is 78 ± 177 m (mean ± standard deviation), deeper than IBCSOv2 (Table S1). We find a positive anomaly near Conger Ice Shelf that cannot be resolved and had to be masked out and interpolated.

West (15,666 $\hbox {km}^{2}$) (R14) has few MBES data (Fig. 1, Fig. S7n). We find a cavity 300–600 m deeper below West N.and West S., respectively (Fig. S7n). On the continental shelf, previously unknown deep troughs at 1400 m depth extend to the edge of the continental shelf. The bathymetry is 106 ± 186 m deeper on average (Table S1).

Amery (R15) is deeper than IBCSOv2 about 200 km from the grounding line but within 2 ± 168 m on average (Table S1). Few observational constraints are available at the grounding line, which has been shown to be too deep^51^. The 1000-m step at Mellor Glacier is largely an artefact. We fit seismic data within 9 ± 37 m, but uncertainties are unresolved near the grounding line (Fig. S7o).

Wilma–Robert/Edward VI and Rayner-Thyer in R16 are 450–300 m deeper than IBCSOv2 (Fig. S7p) and 195 ± 220 m deeper on average (Table S1). Prince Harald (5392 $\hbox {km}^{2}$) and Shirase (821 $\hbox {km}^{2}$) (R17) are 100 and 300 m deeper, respectively. Two previously unknown troughs appear on the continental shelf, with a depth of 1250 m at the shelf break (Fig. 2, Fig. S7q). The bathymetry is 41 ± 176 m deeper on average.

Baudouin (32,952 $\hbox {km}^{2}$), Borchgrevink (21,580 $\hbox {km}^{2}$), Lazarev (8519 $\hbox {km}^{2}$) and Nivl (7285 $\hbox {km}^{2}$) (R18) in Queen Maud Land are 117 ± 189 m deeper than IBCSOv2 (Table S1). The largest changes are found on the eastern part. We fit seismic data^52^ within −57 ± 57 m. The inversion in^53^ is comparable to our product in the eastern part of Baudouin, but shallower elsewhere (Fig. S7r). We lack bathymetric constraints at the edge of the continental shelf, where positive gravity anomalies associated with the geologic transition between the continental shelf front and the deep ocean yield higher uncertainties for the inversion.

Vigrid, Fimbul (40,843 $\hbox {km}^{2}$), Jelbart (10,844 $\hbox {km}^{2}$), Atka, Ekstrom (6872 $\hbox {km}^{2}$) and Quar in R19 are deeper to the north (Fig. S7s)^54^. We find a deeper channel beneath Fimbul at 1570 m depth 20 km from the grounding line (Fig. 3). Jelbart has multiple troughs and a basin at 1200 m depth that includes ridges and pinning points (Fig. 3). Ekstrom is 200 m deeper than IBCSOv2^54^. We fit seismic data within −3 ± 32 m. The bathymetry is 56 ± 136 m deeper on average (Table S1).

Riiser-Larsen (43,450 $\hbox {km}^{2}$) and Brunt–Stancomb Wills (36,894 $\hbox {km}^{2}$) (R20) are challenging to invert. A rise in gravity anomaly between the two ice shelves is difficult to correct, resulting in a lower precision for the inversion (Fig. S7t). We find two channels at 1500 m depth beneath Brunt, consistent with^17^, but 300 m deeper along the main trough and 400 m deeper in the eastern part. We match seismic data within −3 ± 48 m. The bathymetry is 52 ± 144 m deeper on average (Table S1).

For Ronne–Filchner (443,140 $\hbox {km}^{2}$) in R1, we fit seismic data within −3 ± 32^39^. The bathymetry is 19 ± 78 m deeper on average (Table S1), with most differences near the grounding line of Evans (400 m), Hercules (970 m) and Rutford (380 m) (Fig. S7a).

## Discussion

The overall change in bathymetry compared to IBCSOv2 is estimated with seafloor depth 69 ± 159 m deeper on average, with much higher local differences. This magnitude change illustrates the level of uncertainty of existing maps, especially for ice shelf cavities. In the absence of observational constraints, mathematical extrapolations have produced smooth, shallow bathymetric solutions with no troughs. Improvements result from the combination of BMv3.7 on land, MBES/SBES, seismic, AUV, MEOP and CTD data. The 3D inversion maximizes the use of AntGG2021, which has no gaps and a higher spatial resolution than AntGG2016^16^. The AntGG2021 compilation is an improved version of ANTGG2016, with additional gravity data and the use of a better optimized RCR method to reconstruct data gaps. The low-pass filtering of the gravity misfit helps constrain the inversion by removing outliers caused by poorly digitized radar sounding data, excessively localized SBES, and rapid bed transition not constrained by in-situ data (Fig. S3). This critical step is region dependent and varies among prior studies^16,19,20,54^, which makes it difficult to quantify and explain differences between products. Our map of filtered (corrected from aberrant bathymetric measurements) data sources will help future inter-comparison of gravity inversion methods (Fig. S3). In areas with few direct bathymetric measurements, the inversion misfit exceeds 80 m. This situation is exacerbated at the edge of the continental shelf (R12–R14, R19), where bathymetry might be 100–200 m deeper than represented herein because the positive gravity anomalies at the shelf break translate into anomalously shallow areas^21^. Further in-situ measurements are critical to better constrain the inversions in this zone. In regions that are missing airborne or ground gravity measurements (Fig. S1), bathymetric mapping should be analyzed on a large scale (tens to hundreds of kilometers), rather than at the scale of troughs. In such cases, our results allow for the use of a physical interpolation method between a large compilation of in-situ data available offshore and onshore, thus providing a more reliable estimate than the artifacts produced by mathematical interpolations. However, without high resolution airborne gravimetric data in these regions, the uncertainties will remain high (up to 250 m), which has a direct impact on the modeling of warm water pathways.

In the Peninsula, CDW at $+1.2\,^{\circ }$C at 350 m depth in front of Wilkins is below the grounding line depth, which is not exposed to CDW despite a deeper bathymetry than IBCSOv2 (Fig. 3.1). On George VI, the same CDW is deeper than the depth of grounding lines (300–400 m), which is consistent with its slow rate of retreat despite abundant CDW on the continental shelf (Fig. 3.1).

In West Antarctica, water warmer than $+1\,^{\circ }$C at 300 m depth in front of Venable is likely reaching its deep grounding line, whereas older bathymetry (BedMachine, IBCSO) suggested a shallow cavity (Fig. 3-2). For Abbot, warm water in excess of $+1\,^{\circ }$C below 400 m depth remains deeper than the grounding line depths, hence it is protected from CDW, but the previously unknown trough in the middle of Abbot will facilitate the flow of modified CDW along the Antarctic Coastal Current (AACC), which affects glaciers farther west in Pine Island Bay^55^ (Fig. 3). At Pine Island, warm CDW $>+1\,^{\circ }$C^5^ at 600 m depth faces no major obstacle to reach the grounding line.

In East Antarctica, near Cook and Ninnis, CTD data from 1970–1985^56^ reveal cold waters at $-1.2\,^{\circ }$C, similar to Mertz (Fig. 3-5, 3-6), i.e. no modified CDW. Our bathymetry is deeper than IBCSOv2, revealing new troughs, not well constrained by MBES data. Cook lost its western ice shelf in the 1970s and Ninnis in the 2010’s, hence exposure to CDW may not be ruled out^3^.

CTD data in front of Moscow University and Totten reveal warm waters up to $+1\,^{\circ }$C at 500 m depth (Fig. 3-7). Our bathymetry includes easier pathways for CDW toward Moscow University, but with a lack of observational constraints at the shelf break, which is too shallow. On Totten, warm CDW flows down a narrow passage^57^ to yield high ice shelf melt rates^40,58^ and grounding line retreat^59^.

Water at $+0.1{-}0.5\,^{\circ }$C near Denman between 350 and 1400 m depth should reach its deep grounding line (Fig. 3-9), especially with the new bathymetry, which is consistent with the observed grounding line retreat and ice shelf melt rates^58^. Water at $<1\,^{\circ }$C at 500 m depth near West may expose its grounding line (500–600 m depth) to high melt, but we need new MBES data to constrain the inversion (Fig. 3-10).

For Shirase and Prince Harald, water $>0.5\,^{\circ }$C at 580–800 m depth is above the grounding line depth. The new bathymetry reveals a broader, deeper channel on the continental shelf toward both glaciers (Fig. 3-11). On Baudouin, the ice shelf cavities are deeper than IBCSOv2. Water at $+0.25\,^{\circ }$C below 250 m may reach the grounding lines at several locations, but additional observations are needed at the shelf break (Fig. 3-12).

Similarly warm water at the shelf break of Borgrevink may reach its deep grounding line (Fig. 3.13). Fimbul and Jelbart grounding lines at 1100 and 900 m depth are protected from warm water at the shelf break, which is shallow. In contrast, Jelbart, with a trough 750 m deeper than IBCSOv2, is likely exposed to warm waters (Fig. S7t). From Princess Astrid to the Brunt Coast, water at $+0.5\,^{\circ }$C below 900 m is deeper than the shelf break at 450–500 m, which likely protects the ice shelves (Fig. 3-16). The cavity beneath Brunt–Stancomb is deeper than in IBCSOv2. Warm water at 800 m depth stands below the edge of the continental shelf (400–600 m depth).

Several sectors await new observational constraints to resolve pathways for warm water, especially East Antarctica. The AntGG2021 bathymetry provides guidance on the likely location of troughs, sills, and areas of importance to be surveyed, e.g. Cook, Ninnis, Moscow, Shackleton, and West ice shelves. More in-situ observational constraints are needed to refine the results, especially at the shelf break. Ultimately, denser MBES/seismic or AUV coverage, including ice shelf cavities, is desired to improve vertical precision by one order of magnitude and spatial resolution (meters instead of km). Knowing the depth of troughs, sills, and pathways, at that precision will allow to model seasonal to interannual changes in the depth of the thermocline^60^. In the meantime, the AntGG2021 bathymetry should allow an improvement in our characterization of ocean circulation on the continental shelf and ice shelf cavities, which are now fully consistent based on a physical model. The results will be of immediate use to improve the projections of ice mass loss from Antarctica in a warming climate.

## Conclusions

We present a novel, comprehensive bathymetry of Antarctica with all ice shelf cavities, including a smooth transition toward BMv3.7 on land and IBCSOv2 at sea. The results reveal deeper continental shelf in numerous regions, previously unknown troughs, and a greater exposure of the Antarctic grounding line to warm waters. The new product will allow for improvements in future in the modeling of ice-ocean interactions in Antarctica and in turn the reliability of projections of sea level rise from these glaciers because it provides a more realistic, physics-based description of ice shelf cavities than prior products. In the most critical sectors for sea level rise, we recommend detailed MBES surveys, especially at the shelf break, to improve the products for ice sheet/ocean modeling.

## Supplementary Information

Supplementary Information.

## Data Availability

The products described herein are available on Dryad at https://doi.org/10.5061/dryad.rbnzs7hkc. The AntGG2021 gravity compilation is available on pangea at https://doi.pangaea.de/10.1594/PANGAEA.971238. CTD data is available at the Hadley center and at https://climatedataguide.ucar.edu/, MEOPs data from the MEOPs archive at https://www.meop.net/database/meop-databases/meop-ctd-database.html. Other in-situ bathymetric data are available at https://ramadda.data.bas.ac.uk/repository/entry/show?entryid=a72a50c6-a829-4e12-9f9a-5a683a1acc4a and at https://ds.iris.edu/mda/20-017/.
