# Supplementary material for: Bathymetry of the Antarctic continental shelf and ice shelf cavities from circumpolar gravity anomalies and other data
Source: Sci Rep. 2025 Jan 7;15:1214. doi: 10.1038/s41598-024-81599-1 (PMC11707401; doi:10.1038/s41598-024-81599-1)
Supplement: Supplementary file 1 — Supplementary Information. [file 41598_2024_81599_MOESM1_ESM.pdf]

Supplementary material for Bathymetry of the Antarctic continental shelf and ice shelf cavities from circumpolar gravity anomalies and other data

Raphaelle Charrassin[1,2], Romain Millan[2,*], Eric Rignot[1,2,3,4], and Mirko Scheinert[5]

[1]Department Earth System Science, University of California Irvine, Irvine, 92697 CA, USA.
[2]Université Grenoble Alpes, CNRS, IRD, INP, 38400, Grenoble, Isère, France
[3]Jet Propulsion Laboratory, California Institute of Technology, Pasadena, 91107 CA, USA.
[4]Department Civil and Environmental Engineering, University of California Irvine, Irvine, 92697 CA, USA.
[5]TUD Dresden University of Technology, Chair of Geodetic Earth System Research, 01062 Dresden, Germany.
[*]romain.millan@univ-grenoble-alpes.fr
[+]these authors contributed equally to this work

ABSTRACT

Bathymetry critically influences the intrusion of warm Circumpolar Deep Water onto the continental shelf and under ice shelf cavities in Antarctica, thereby forcing ice melting, grounding line retreat, and sea level rise. We present a novel and comprehensive bathymetry of Antarctica that includes all ice shelf cavities and previously unmeasured continental shelf areas. The new bathymetry is based on a 3D inversion of a circumpolar compilation of gravity anomalies constrained by measurements from the International Bathymetric Chart of the Southern Ocean, BedMachine Antarctica, and discrete seafloor measurements from seismic and ocean robotic probes. Previously unknown troughs with thicker ice shelf cavities are revealed in many parts of Antarctica, especially East Antarctica. The greater depths of troughs on the continental shelf and ice shelf cavities imply that many glaciers are more vulnerable to ocean subsurface warming than previously thought, which may increase the projections of sea level rise from Antarctica.

Contents of this file

1. Text S1
2. Table S1 to S3
3. Figures S1 to S11

Introduction

This document contains specific explanations about the remove-compute-restore method used to obtain the consistent free-air anomaly gravity grid ANTGG2022 (Text S1), Fig. S1-S6 for masks, data sources, and evaluation; Fig. S7a-t for 20 regions, Fig. S8 for CTD data in Fig. 3, Fig. S9 for the inversion uncertainty elevation map and Fig. S10 and S11 for the comparison of the ANTGG2022 bathymetry and Bedmap2 and BedMachine.

Text S1.

Description of the RCR method and the background model

The remove-compute-restore (RCR) method in geodesy helps align different gravity data sets using an a-priori Earth gravity model based on satellite data and, optionally, topographic data(1). The RCR technique (2) comprises three steps. In the "remove" step, long-wavelength and short-wavelength signal parts are removed from the original gravity observations using a-priori model, in this case the high-resolution model SATOP-1 (3). In the "compute" step all desired gravity-field related quantities are computed as functionals of the disturbing potential T using least-squares collocation (LSC, see below). In the "restore" step the information from the a-priori model SATOP-1 is restored for the long-wavelength and short-wavelength signal parts in terms of the respective functionals.

The remove step of the RCR procedure accounts for biases between individual surveys and for long-wavelength signal parts, which cannot be resolved over a limited areas as given by the individual gravity surveys, as well as considers short-wavelength signal parts which are mainly generated by topography. The latter reduction leads to a smoothing of the data, which facilitates interpolation and prediction. To perform this reduction, a high-resolution a-priori model is constructed based on the satellite-only model GOCO-05S (4) and the EARTH2014 topographic model (5). The method to combine the information of these two models is based on a regularization of the satellite normal equation using the topographic model as a-priori information. The combination itself is performed in the spheroidal-harmonic domain, resulting in the model SATOP-1 of up to harmonic degree and order 5480 (3). Then, the necessary evaluation of this background model is done on a 3D grid that covers a latitude range from 60° to 90°S (at 1' resolution), a longitude range from 0° to 360° (at 2' (60 to 75.4°S) and 4' (75.4 to 90°S) resolution) as well as a vertical range from -1 to 6 km (at 200 m resolution). At this grid, the disturbing potential, T, and its functionals (like gravity anomaly) are calculated. To infer the respective quantities for the remove step (e.g. at specific data points) and the restore step (at points of the final 2D grid at different specific height levels), respectively, a suitable interpolation scheme is applied using this pre-calculated 3D grid. Zingerle et al. (3) also discuss the strategy to evaluate and validate ground-based and airborne gravity anomaly data sets, starting with the AntGG2016 compilation (1), using SATOP-1, thereby taking different spectral bands into account.

Description of the least-square collocation method

For the compute step, the method of least-squares collocation (LSC) (2; 6) is applied to the residual observations (which result from the reduction using the a-prori model SATOP-1 in the remove step). LSC provides a prediction method solely based on the stochastic properties of the input data. An advantage is that all needed functionals of the disturbing potential, T, are inferred at any given point in space (hence, at a regular grid with desired resolution as well as at different height levels), and that also accuracy measures are provided. To circumvent a major problem of LSC, namely the set-up and inversion of the covariance matrix of the (residual free-air gravity anomalies) observations, which has the dimension of the number of observations squared, a localization of the covariance function is introduced. This step is possible since the disturbing potential T as harmonic function is defined at or close to a sphere (7), leading to locally dominating homogeneous-isotropic covariance functions. Similar to the technique described above, the covariance values are pre-calculated at an intermediate regular grid to facilitate simple and fast interpolation algorithms. For the details of this procedure and further approximations see (7). Furthermore, an optimal partitioning of the entire collocation region (hence, Antarctica as a whole) into sub-regions is realized, which significantly reduces computation time and memory requirements (7). This step is accomplished taking a certain regional overlapping into account as well as minimizing fringe effects. The entire method, called partition-enhanced LSC (PE-LSC), is elaborated in detail by (7) and validated using data sets from Antarctica.

Region	vs IBCSOv2, mean±Std dev (m)	max/min (m)	vs seismic, mean±Std dev (m)
R1 Weddell	-19±77	761/-973	-3±32
R2 Bowman	-98±176	851 /-1276	-2±14
R3 English	-58±134	600 /-796	4±43
R4 Eights	-77±173	791 /-1146	N.A.
R5 Walgreen	-47±113	307 /-433	1±38
R6 Philbin	-107±99	199 /-300	TBD
R7 Bakutis	-16±98	723 /-409	N.A.
R8 Saunders	-98±196	727 /-907	N.A.
R9 Ross	-25±101	1659 /-1434	0±100
R10 Victoria Land	-70±203	656 /-1063	N.A.
R11 George V	-73±171	955 /-991	N.A.
R12 Sabrina	-73±230	1047 /-2411	37±81
R13 Queen Mary	-78±177	835 /-1163	N.A.
R14 Leopold	-106±186	622 /-996	N.A.
R15 Amery	-22±168	877 /504	9±37
R16 Mawson	-195±221	607 /-1228	N.A.
R17 Prince Harald	-41±176	749 /-1002	N.A.
R18 Princess Ragnhild	-117±190	406 /-1237	-57±57
R19 Princess Martha	-56±177	835 /-1163	-3±32
R20 Brunt-Stancomb	-52±143	840 /-853	-3±48

Table 1. Comparison of gravity inversions with IBSCOv2 (with minimum and maximum values) and seismic data.

Region	seismic data	AUV data
R1 Weddell	(8)	N.A.
R2 Bowman	(9)	N.A.
R3 English	(10)	N.A.
R4 Eights	N.A.	N.A.
R5 Walgreen	(11)	(12)
R6 Philbin	N.A.	N.A.
R7 Bakutis	N.A.	N.A.
R8 Saunders	N.A.	N.A.
R9 Ross	(10)	N.A.
R10 Victoria Land	N.A.	N.A.
R11 George V	N.A.	N.A.
R12 Sabrina	(13)	N.A.
R13 Queen Mary	N.A.	N.A.
R14 Leopold	N.A.	N.A.
R15 Amery	(14)	N.A.
R16 Mawson	N.A.	N.A.
R17 Prince Harald	N.A.	N.A.
R18 Princess Ragnhild	(10; 15)	N.A.
R19 Princess Martha	(16)	N.A.
R20 Brunt-Stancomb	(10)	N.A

Table 2. Seismic and AUV data sources.

Region	Mean elevation (m)	Standard deviation (m)
R1 Weddell	147	24
R2 Bowman	136	28
R3 English	139	35
R4 Eights	145	27
R5 Walgreen	97	20
R6 Philbin	106	12
R7 Bakutis	145	53
R8 Saunders	183	58
R9 Ross	201	39
R10 Victoria Land	180	45
R11 George V	223	36
R12 Sabrina	185	59
R13 Queen Mary	153	30
R14 Leopold	148	14
R15 Amery	150	6
R16 Mawson	150	1
R17 Prince Harald	149	7
R18 Princess Ragnhild	147	24
R19 Princess Martha	113	20
R20 Brunt-Stancomb	132	25

Table 3. Inversion uncertainty statistics (meters) calculated for each inversion domain. This was assessed using error assessments on target domains (Fig S4-S6) to scale AntGG2021 standard deviations (Fig 1).

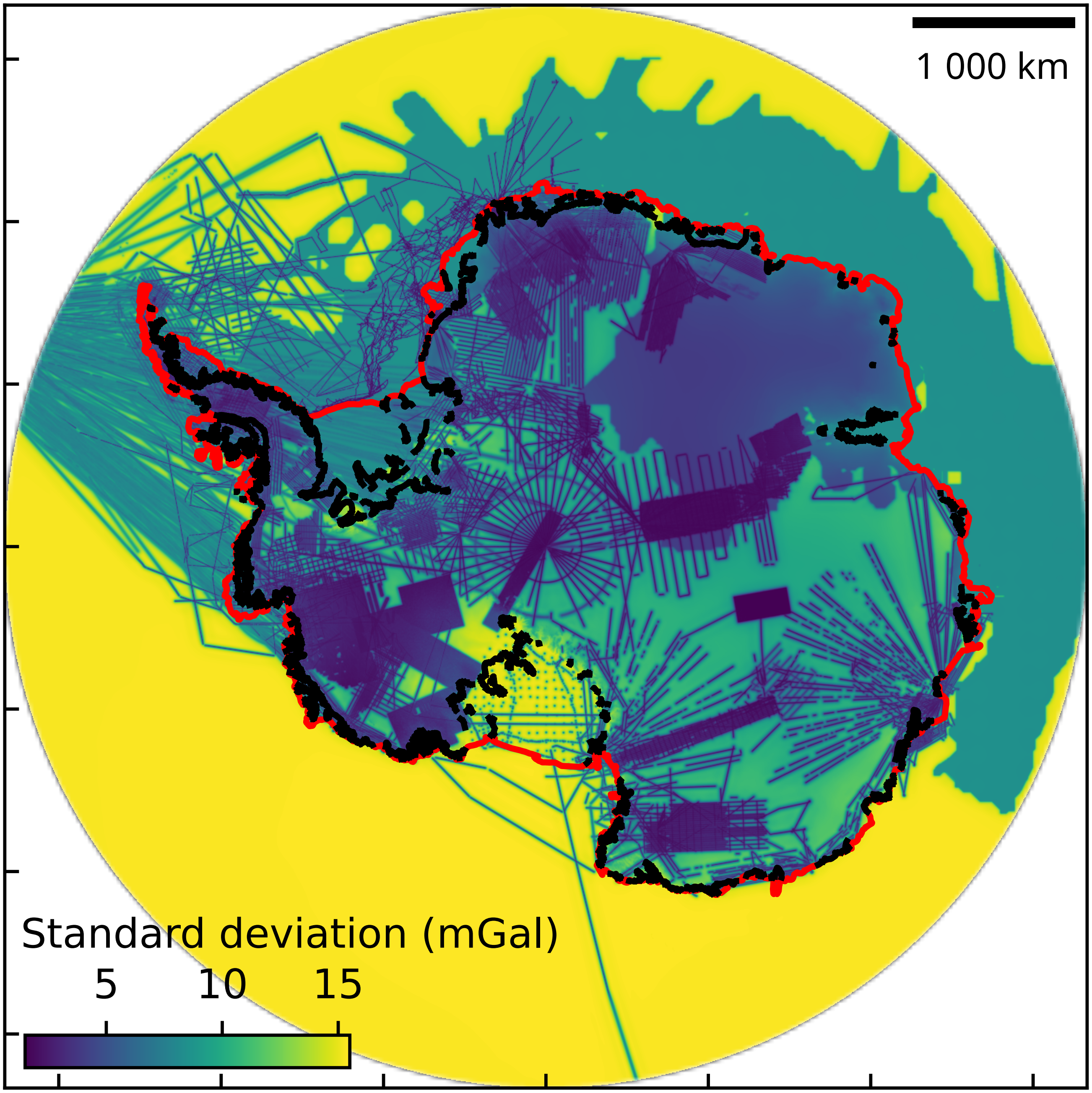

Figure S1. Standard deviation map of free-air gravity anomalies from ANTGG2021. The map is color coded from 1,5 mGal (blue) to +15,5 mGal (yellow).

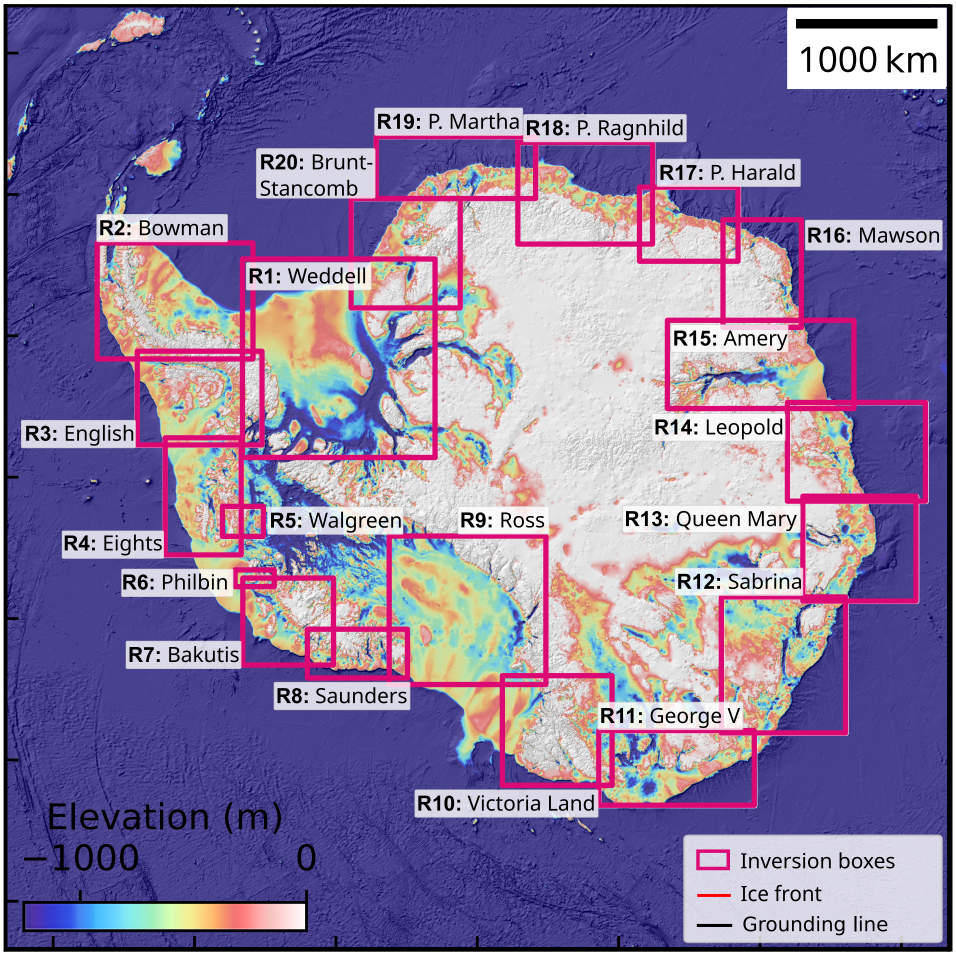

Figure S2. **Inversion mask for the new bathymetry**. The bathymetry is color coded from -1,500 m (blue) to +1,250 m (white). Inversions of ANTGG2021 have been processed over the yellow domains R1-R20. Inversions have been split, named and calculated according to the boxes shown in pink.

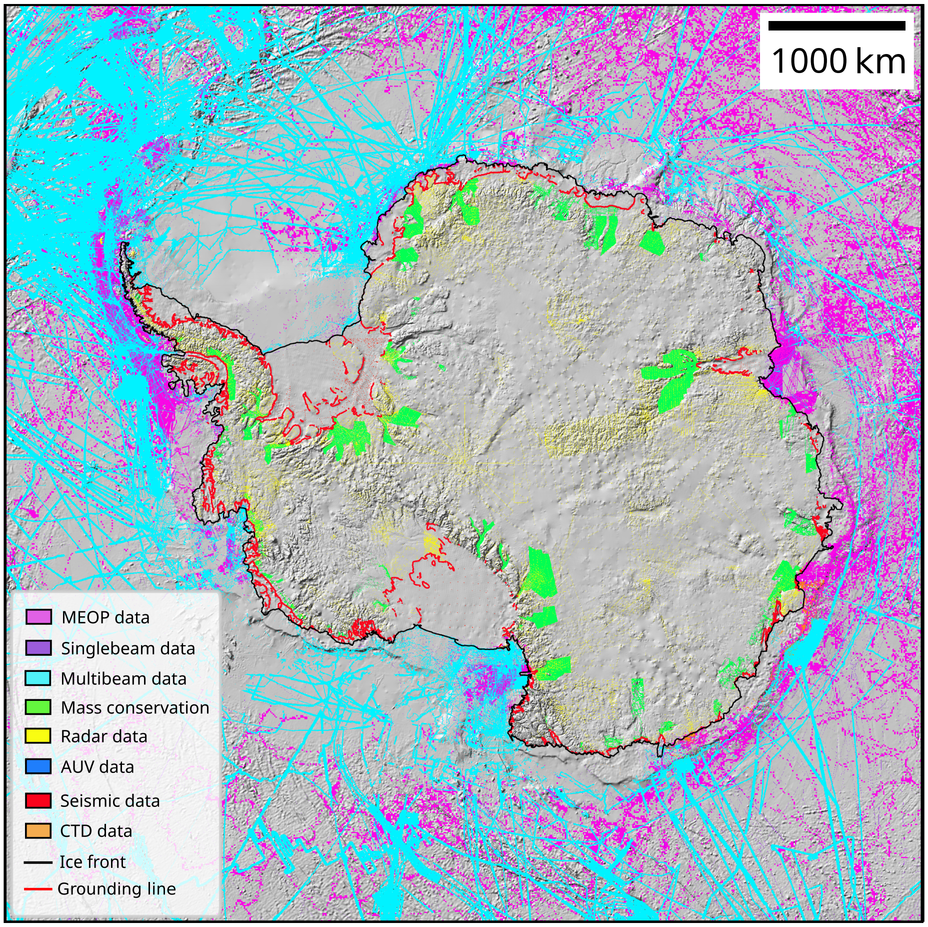

Figure S3. Data sources used to constrain the gravity inversion. MEOP data is pink, single-beam echo sounding (SBES) data is purple, multibeam echo sounding (MBES) data is cyan, bed elevation from mass conservation (BMv3.7) is green, radar thickness data is yellow, Autonomous Underwater Vehicle (AUV) sonar data is dark blue, seismic data is orange, and CTD data is red.

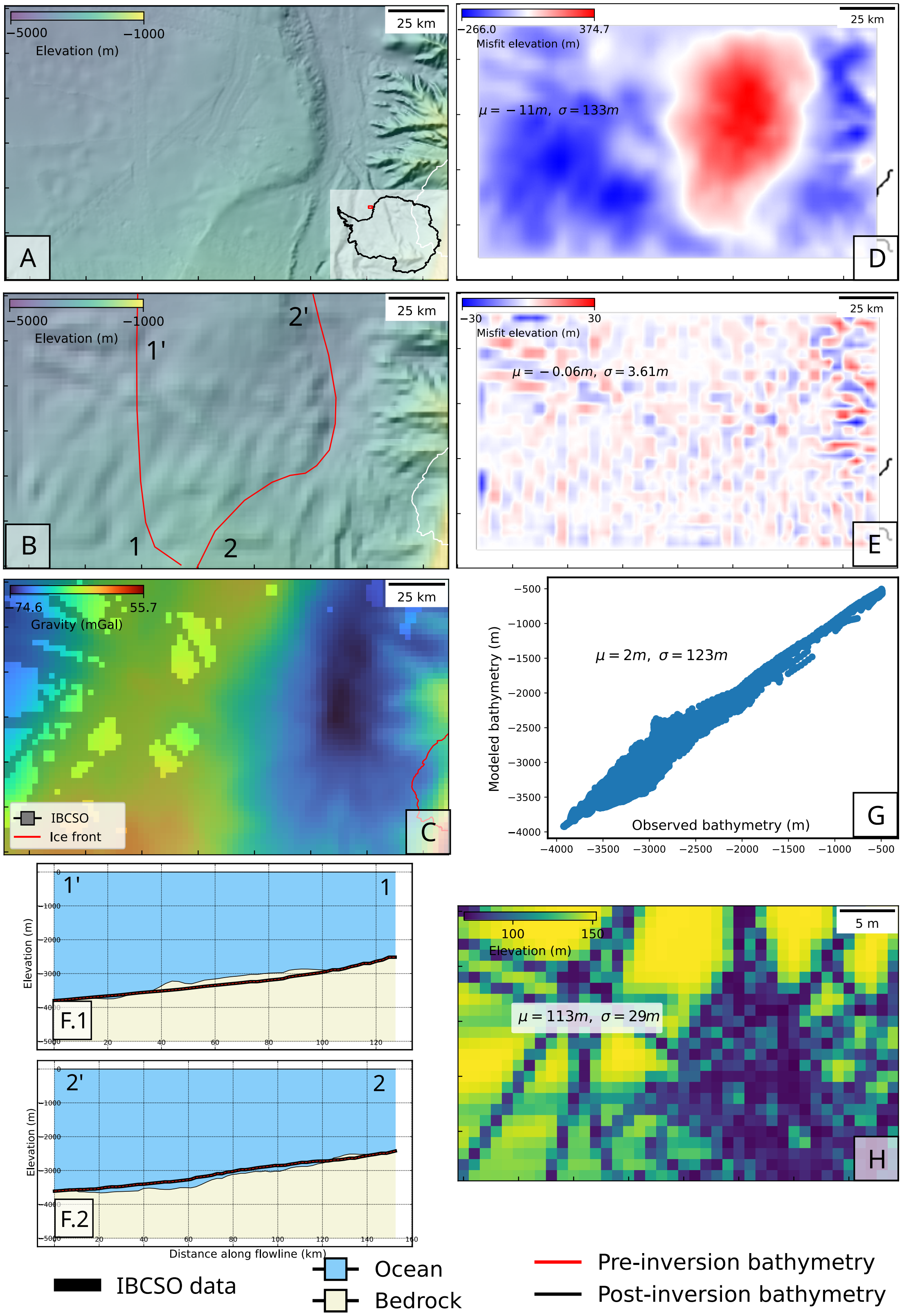

Figure S4. Performance of gravity inversion over a test area in front of Brunt-Stancomb ice shelf. (A) IBCSOv2 color coded from -5000 m (blue) to -1000 m (yellow) with shaded relief (z-factor of 10). (B) New bathymetry and location of profiles 1 (F.1) and 2 (F.2). (C) IBCSOv2 data in grey, and free-air gravity anomalies from ANTGG2021 (mGal). Misfit between initial forward gravity model of topography and ANTGG2021 (D) and new bathymetry forward gravity model (E), converted to equivalent meters. (G) Scatter plot of calculated versus MBES data. (H) Estimate of the elevation uncertainty (m), as a function of the gravity standard deviation.

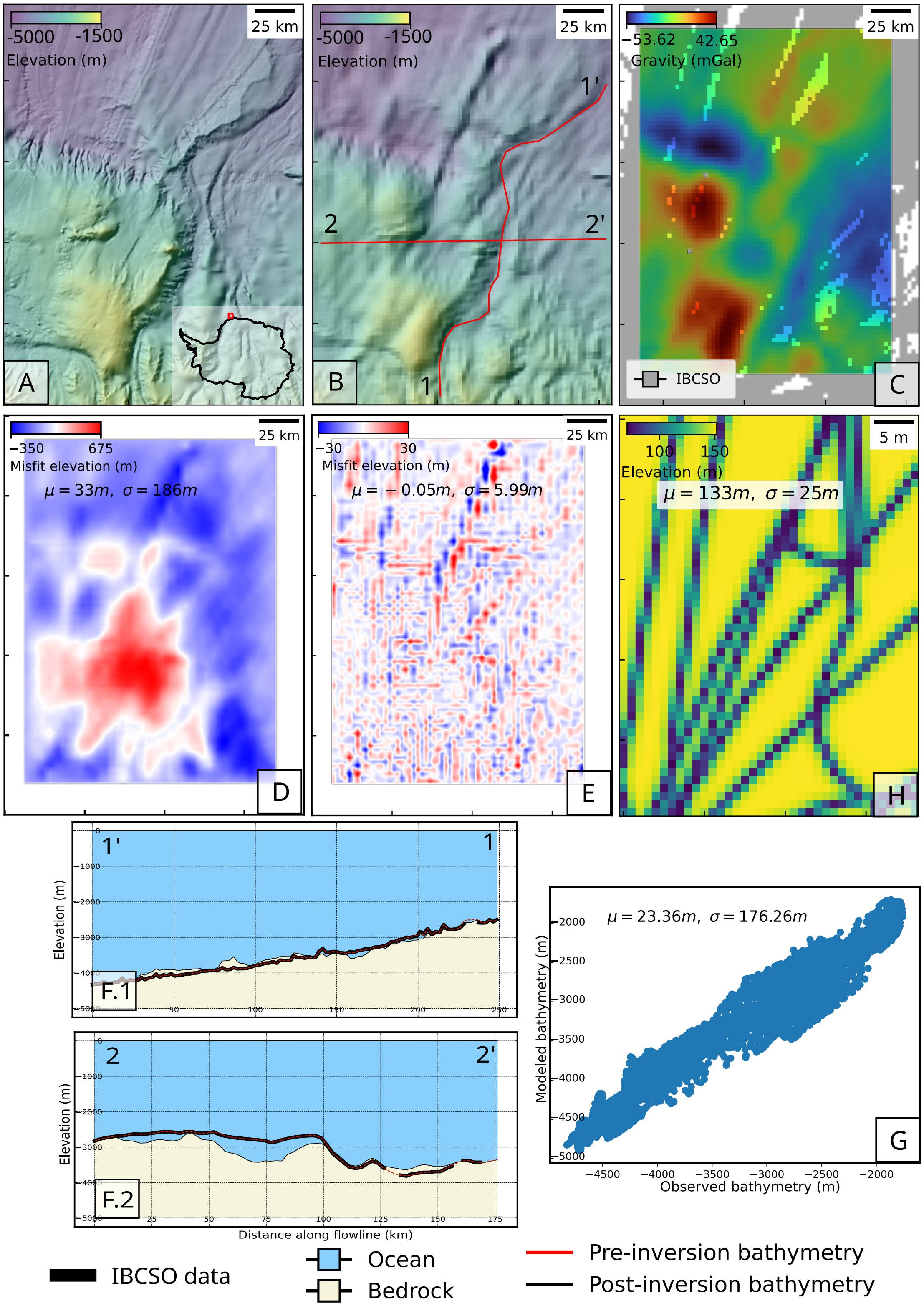

Figure S5. Performance of gravity inversion over a test area in front of Jelbart ice shelf. (A) IBCSOv2 color coded from -5000 m (blue) to -1500 m (yellow) with shaded relief (z-factor of 10). (B) New bathymetry and location of profile 1 (F.1) and 2 (F.2). (C) IBCSOv2 data in grey, and free-air gravity anomalies from ANTGG2021 (mGal). Misfit between initial forward gravity model of topography and ANTGG2021 (D) and new bathymetry froward gravity model (E), converted to equivalent meters. (G) Scatter plot of calculated versus IBCSOv2 data. (H) Estimate of the inversion elevation uncertainty (m), as a function of the gravity standard deviation.

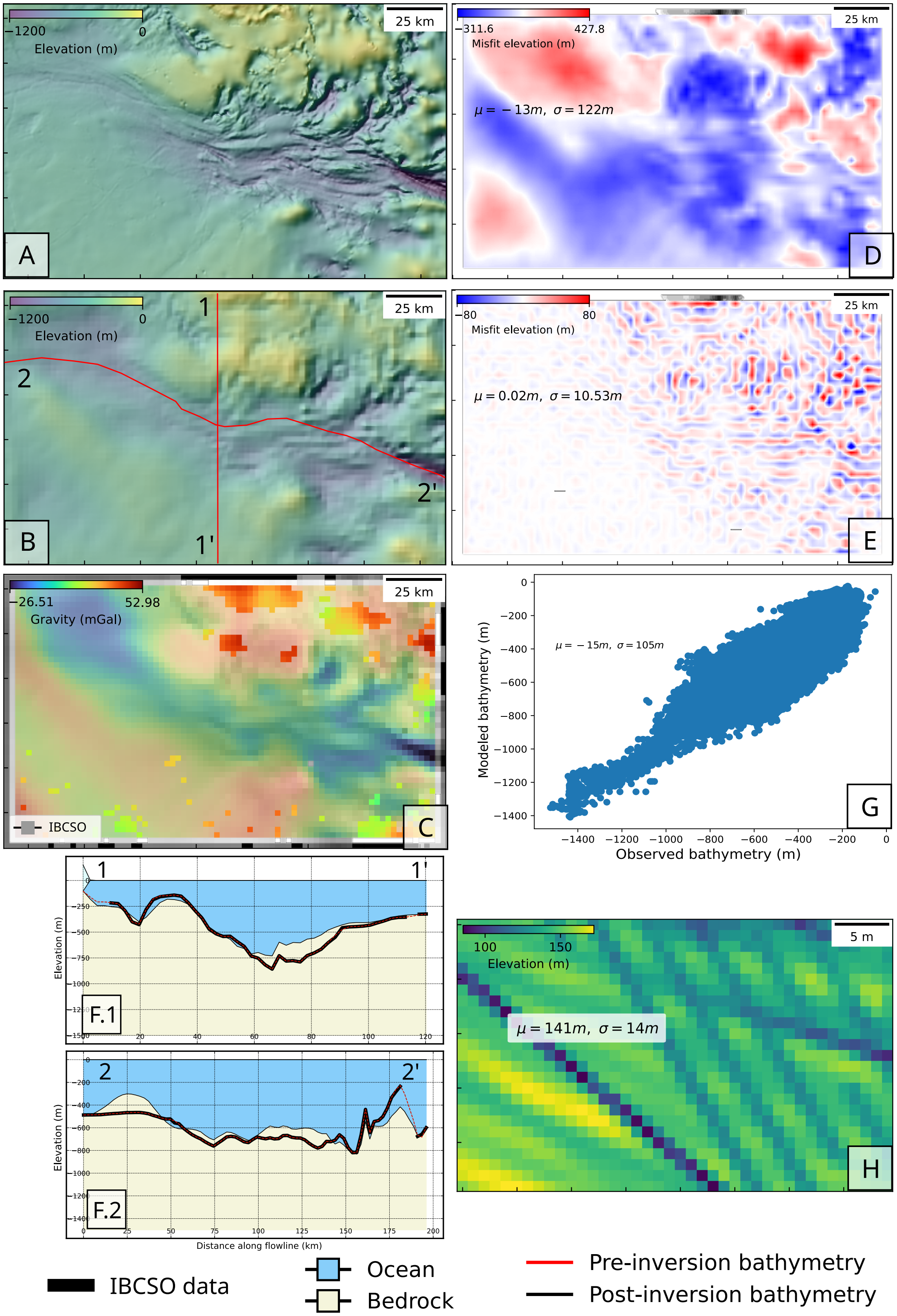

Figure S6. Performance of gravity inversion over a test area in front of George VI ice shelf. (A) IBCSOv2 color coded from -1200 m (blue) to 0 m (yellow) with shaded relief (z-factor of 10). (B) New bathymetry and location of profiles 1 (F.1) and 2 (F.2). (C) IBCSOv2 data in grey, and free-air gravity anomalies from ANTGG2021 (mGal). Misfit between initial forward gravity model of topography and ANTGG2021 (D) and new bathymetry froward gravity model (E), converted to equivalent meters. (G) Scatter plot of calculated versus MBES data. (H) Estimate of the inversion elevation uncertainty elevation (m), as a function of the gravity standard deviation.

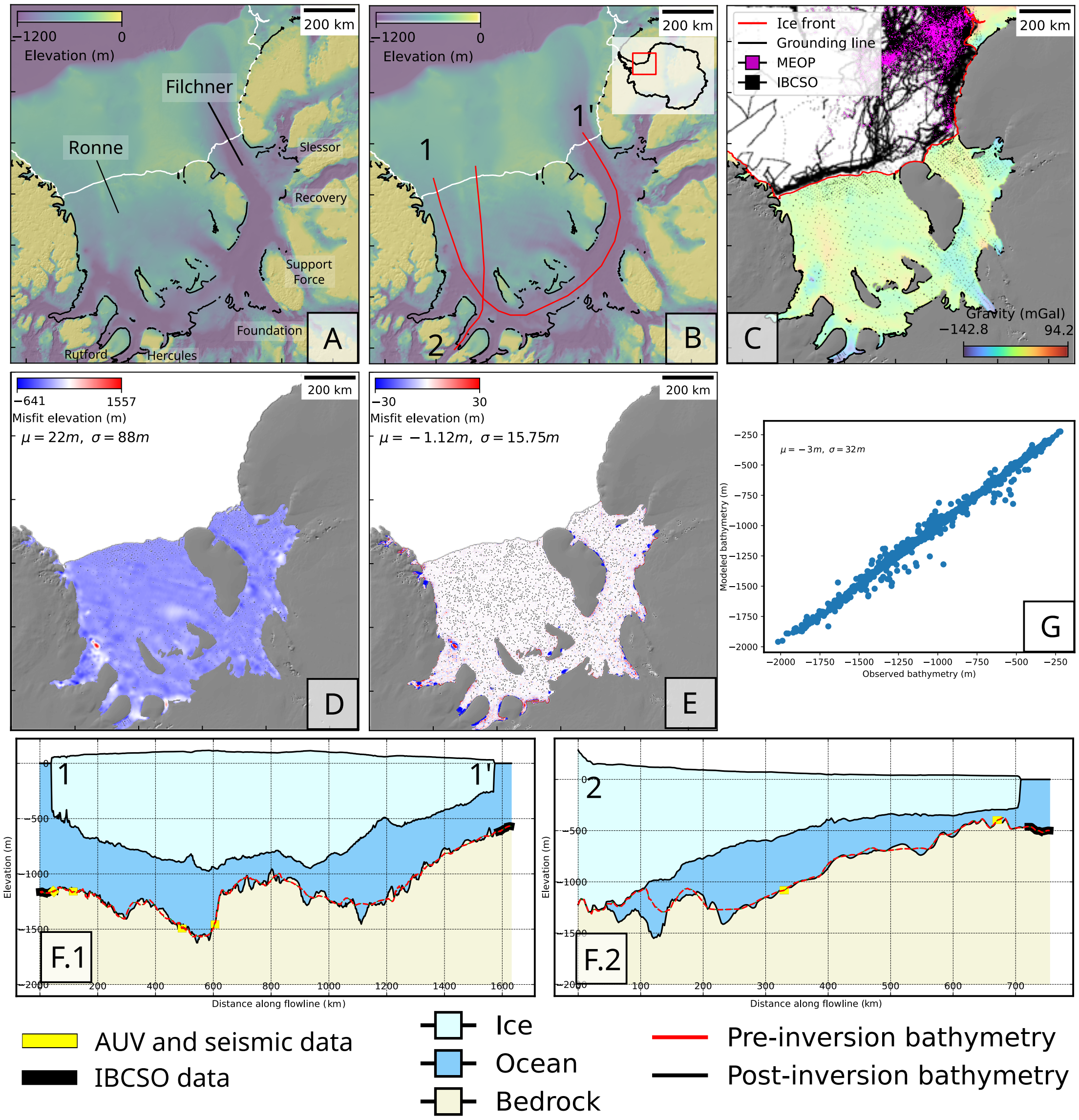

Figure S7a. **Gravity inversion of R1 Ronne, Ronne-Filchner Ice Shelf.**(A) IBCSOv2 color coded from -1200 m (blue) to 0 m (yellow) with shaded relief. (B) New bathymetry and profiles (F.1) to (F.2). (C) MBES data in black and MEOP data in pink. Free-air gravity anomalies (mGal) color coded. Misfit between initial forward gravity model of topography and ANTGG2021 (D) and new bathymetry froward gravity model (E), converted to equivalent meters. (G) Scatter plot between seismic measurements and auv (if data exists) and the new bathymetry.

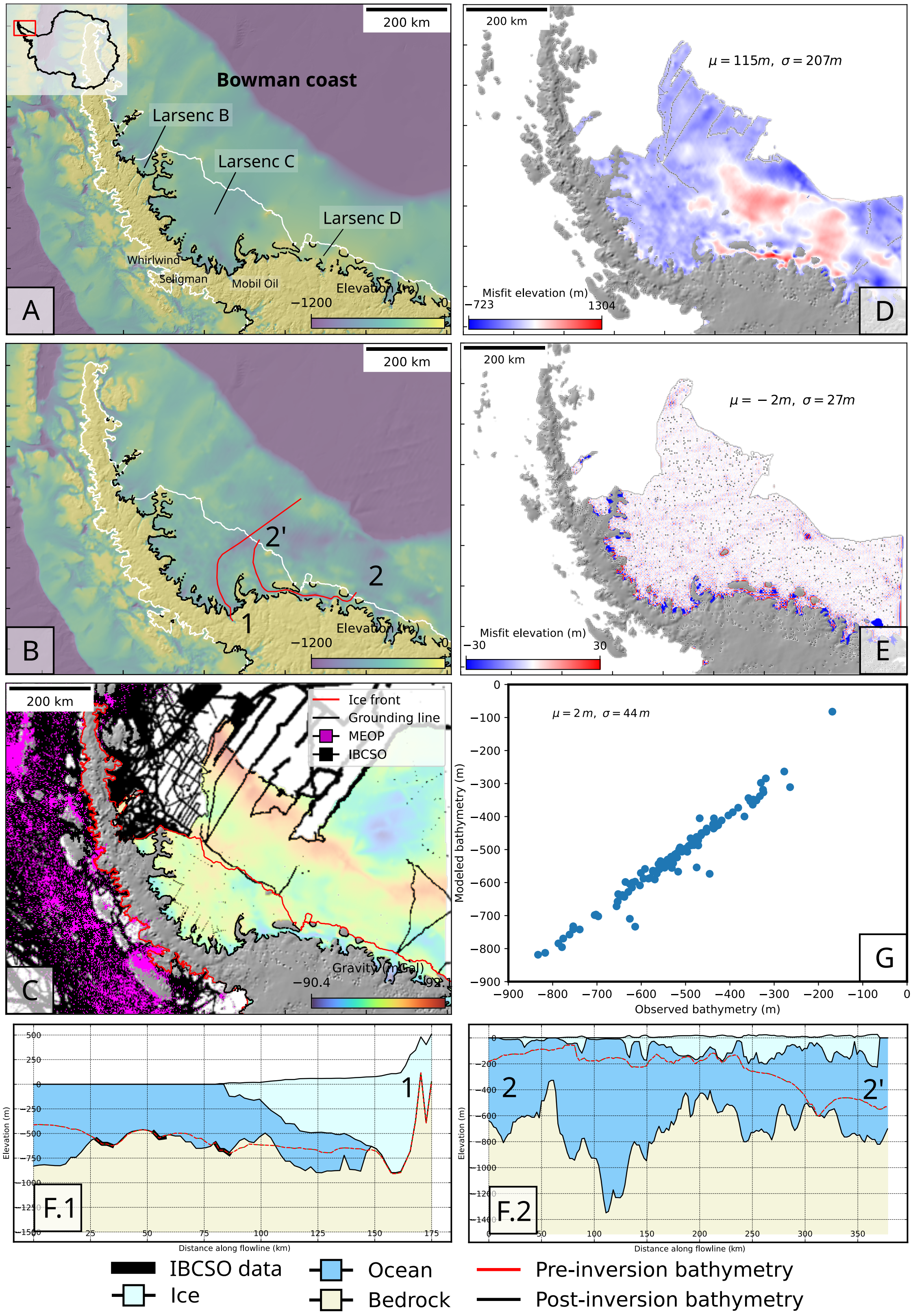

Figure S7b. Gravity inversion of R2 Bowman, Larsen C and D ice shelves.

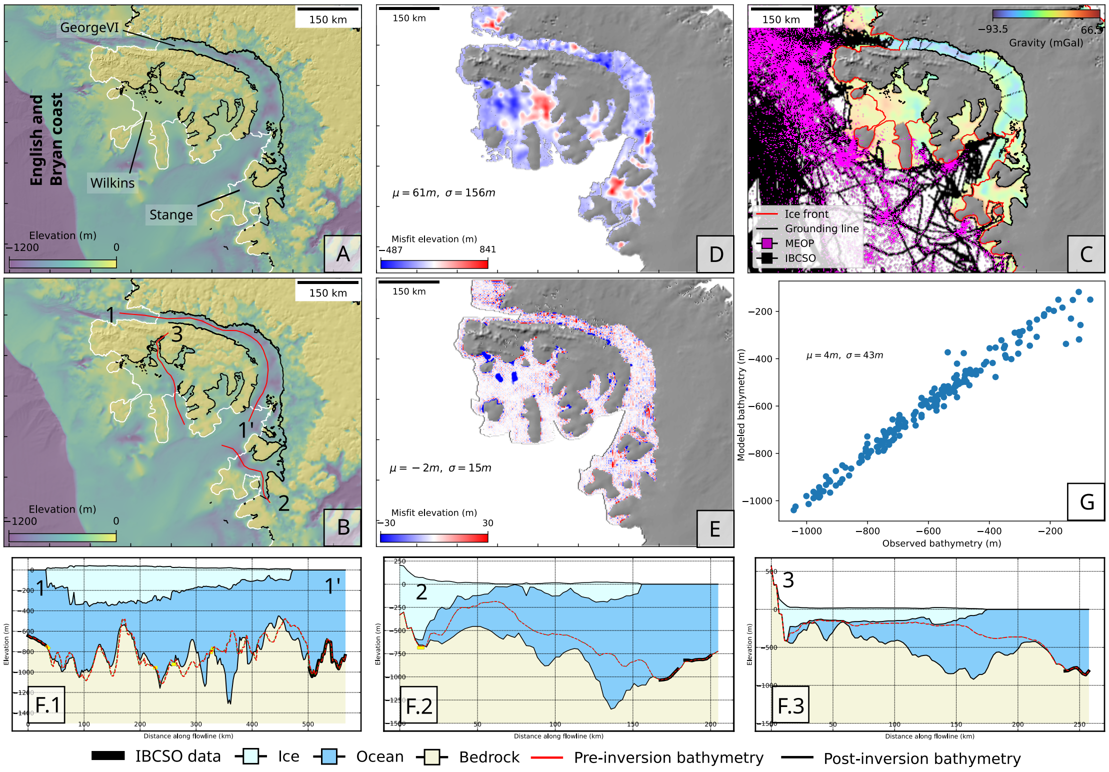

Figure S7c. **Gravity inversion of R3 English, George VI Ice Shelf.**

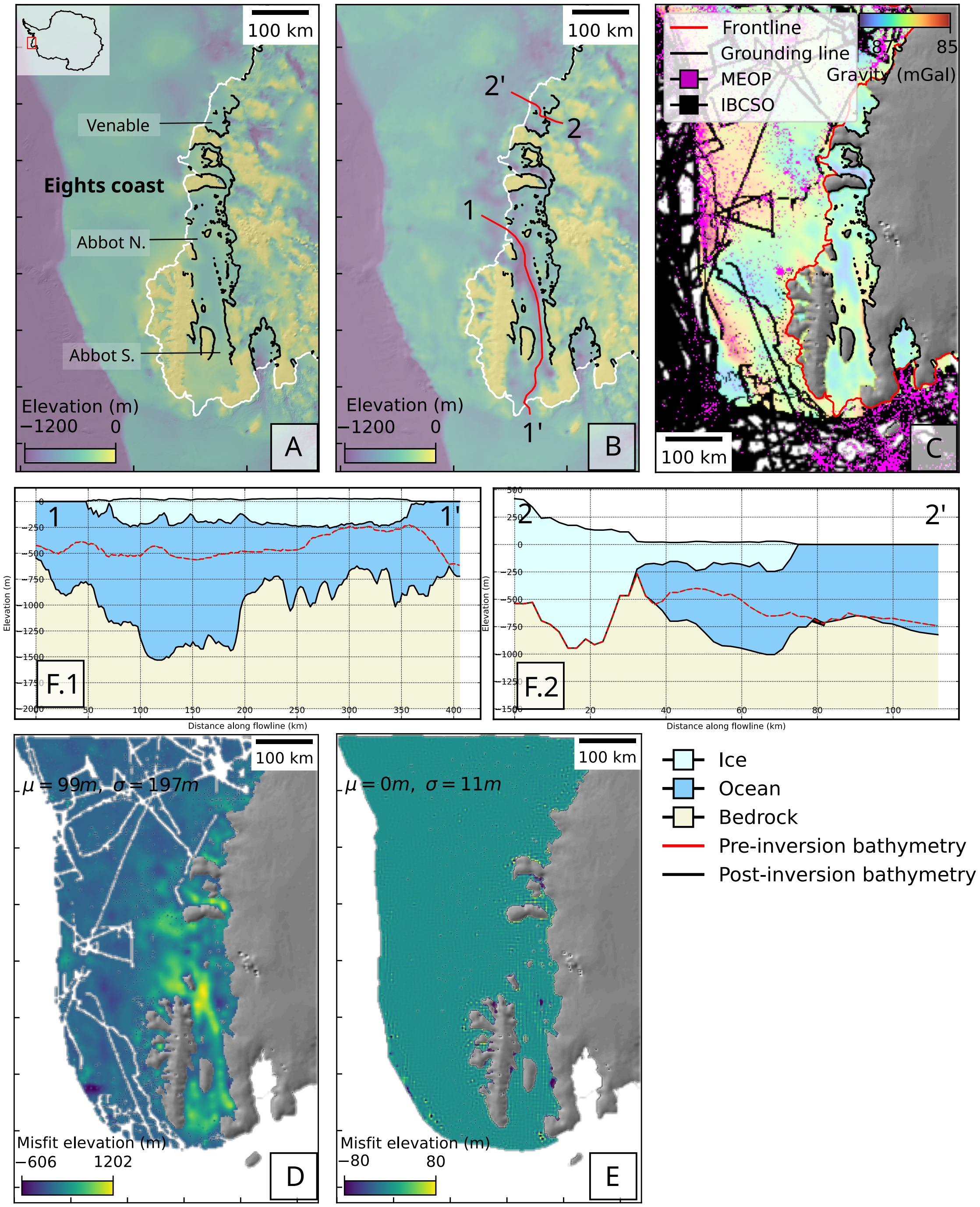

Figure S7d. **Gravity inversion of R4 Eights, Abbot Ice Shelf.**

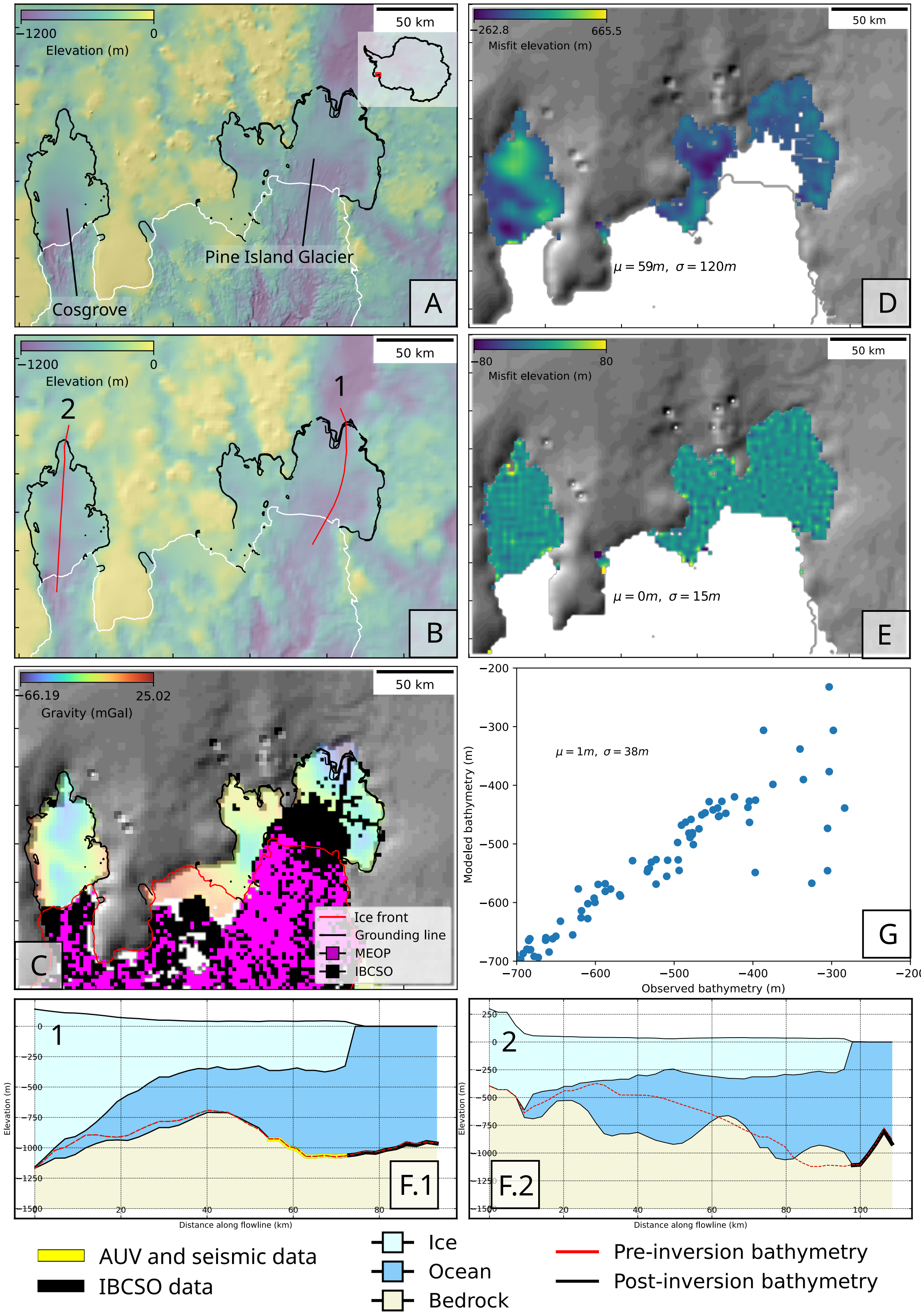

Figure S7e. **Gravity inversion of R5 Walgreen, Cosgrove, Pine Island ice shelves.**

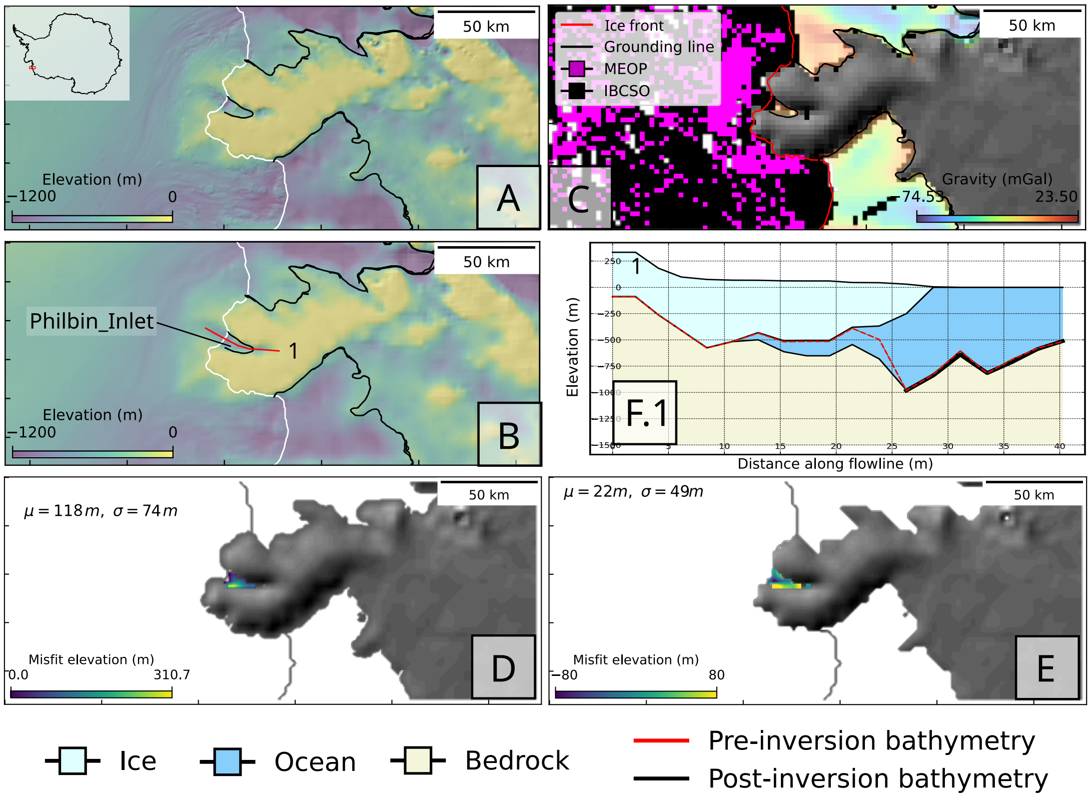

Figure S7f. **Gravity inversion of R6 Philbin.**

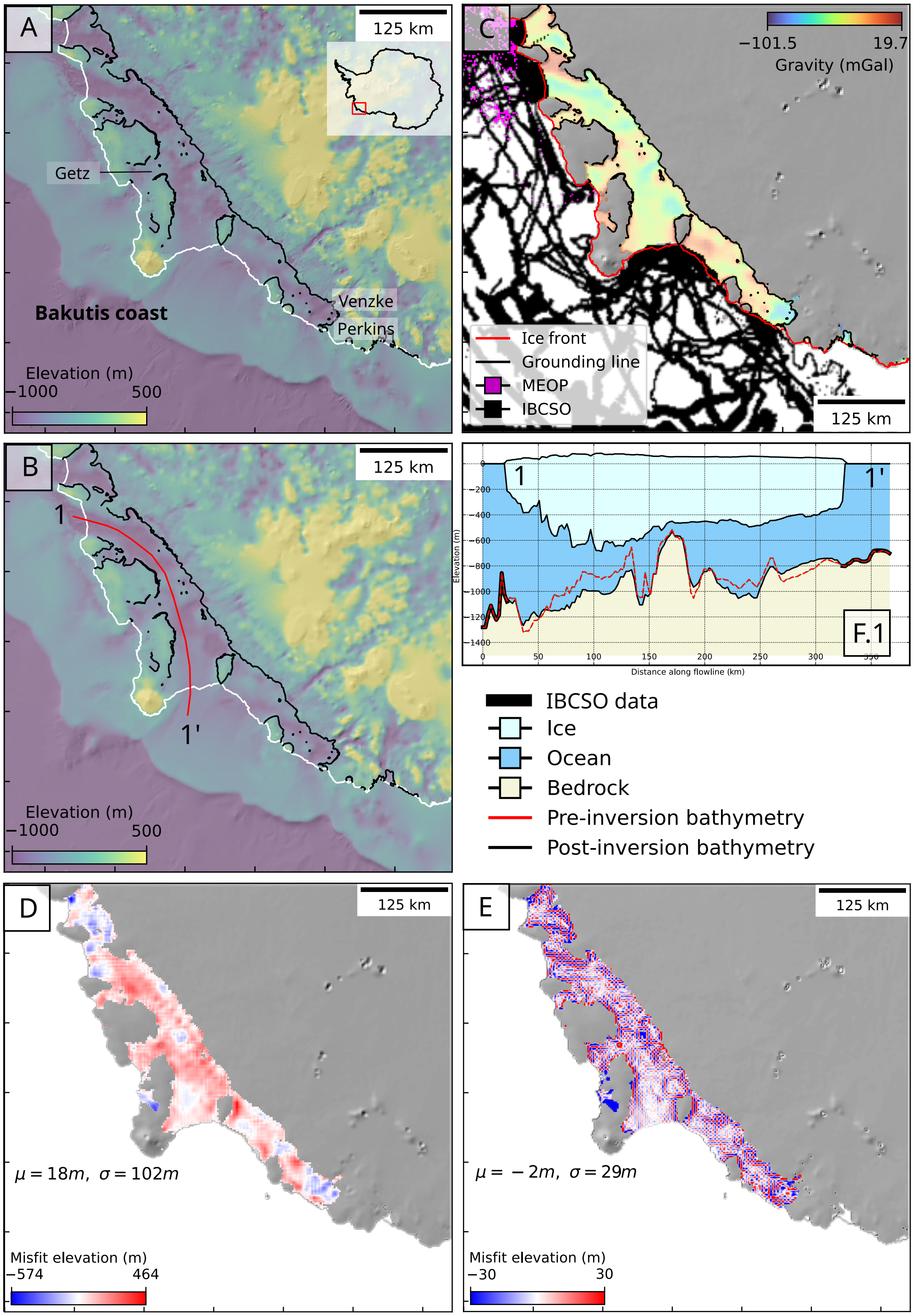

Figure S7g. **Gravity inversion of R7 Bakutis, Getz Ice Shelf.**

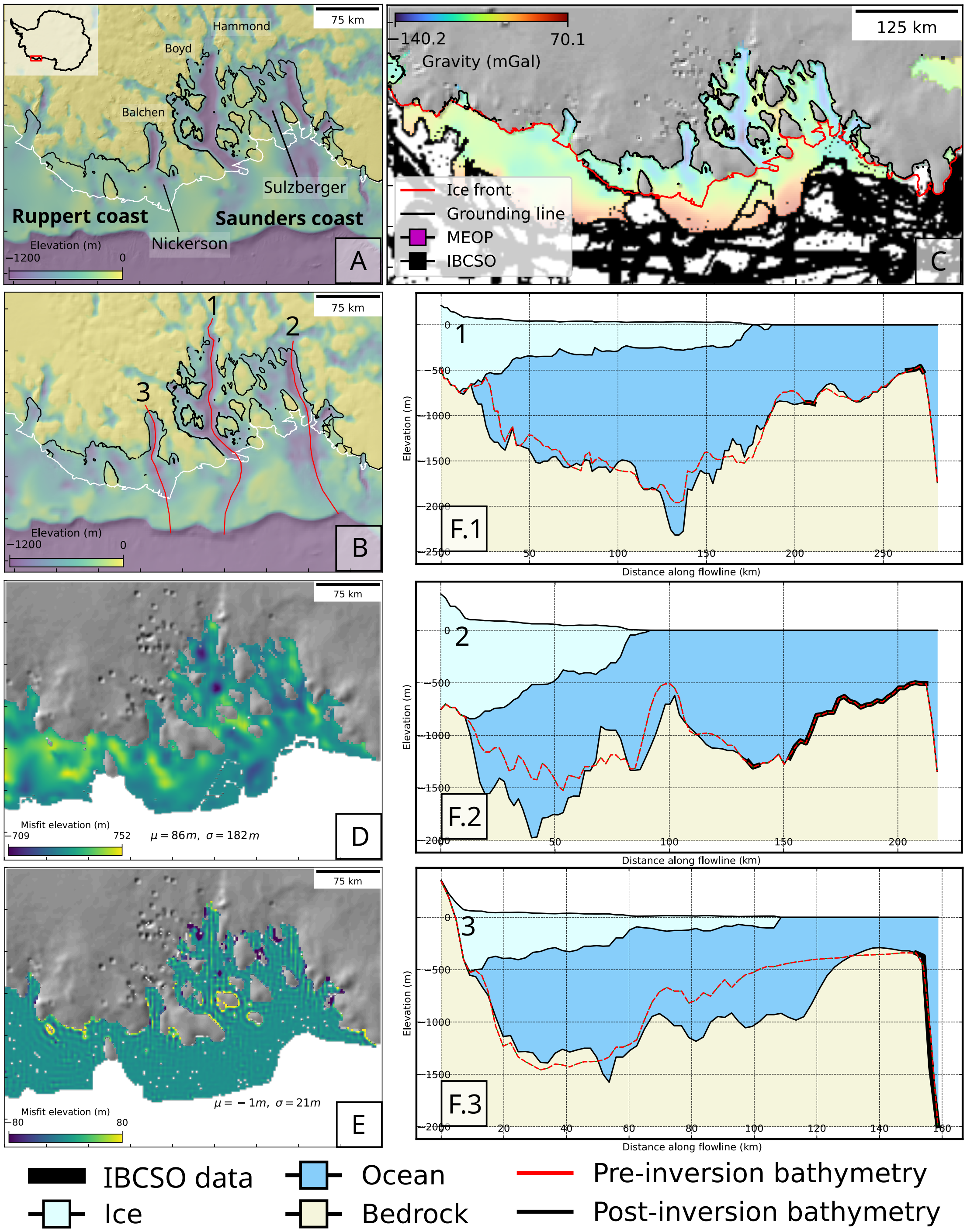

Figure S7h. Gravity inversion of R8 Saunders, Nickerson and Sulzberger ice shelves.

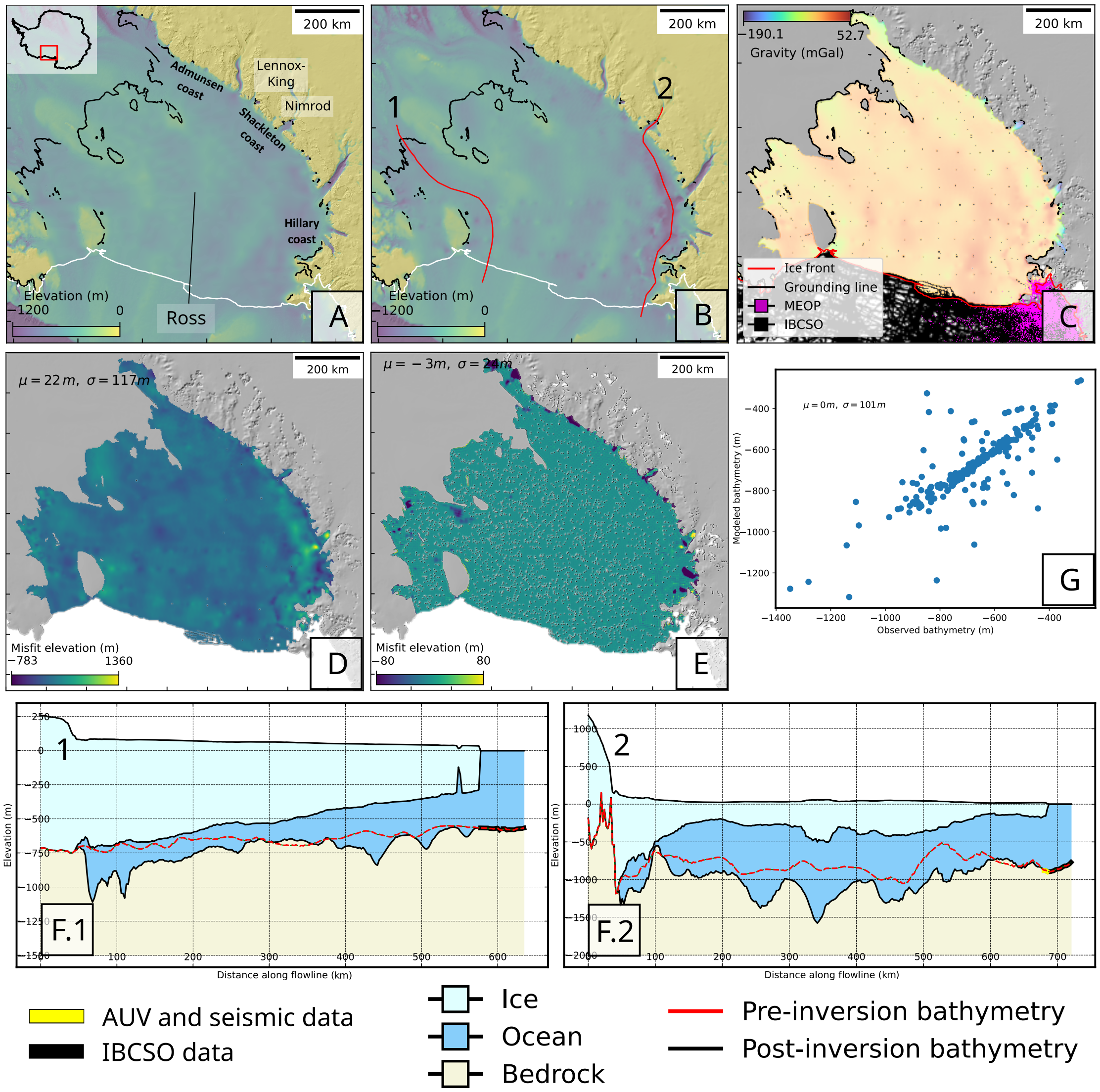

Figure S7i. **Gravity inversion of R9, Ross Ice Shelf.**

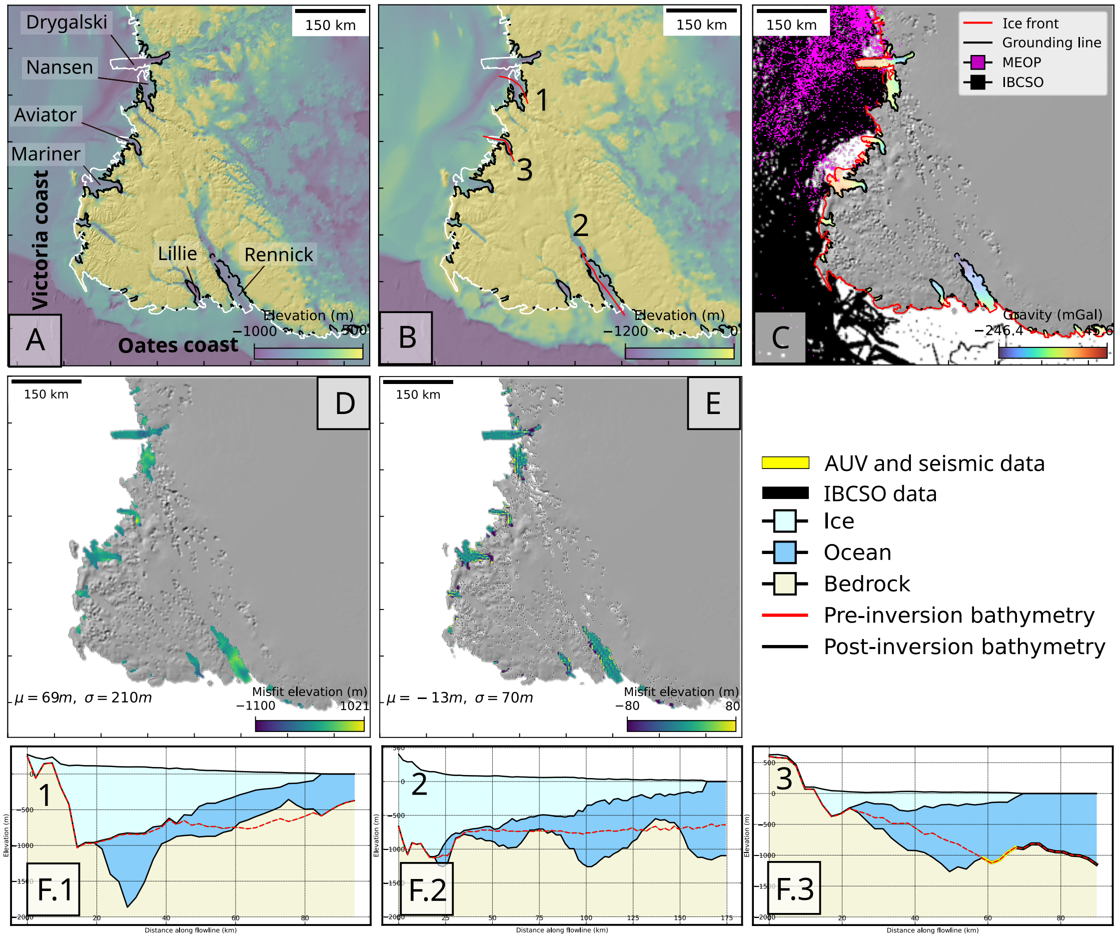

Figure S7j. **Gravity inversion of R10, Victoria Land, Nansen and Rennick ice shelves.**

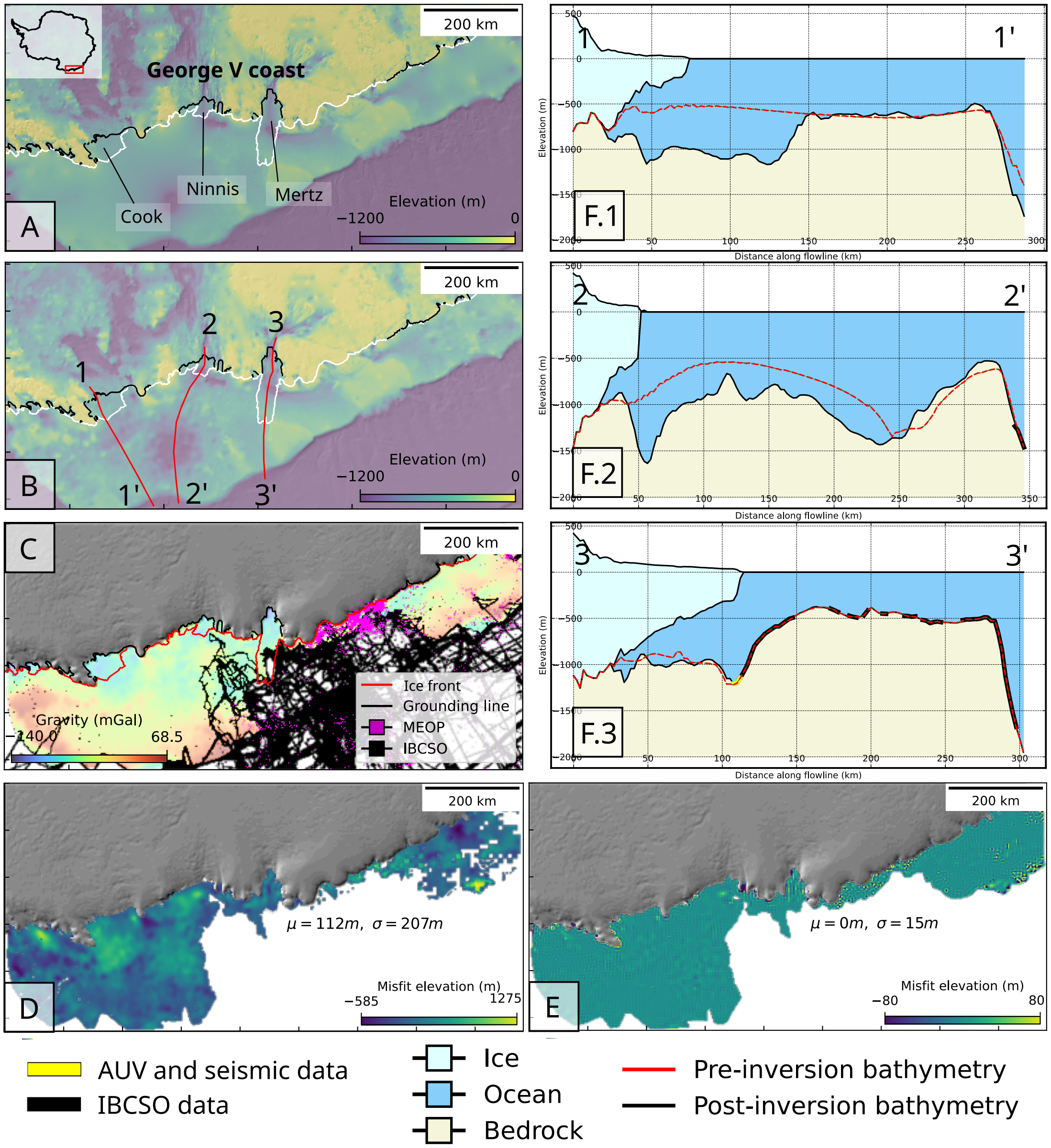

Figure S7k. **Gravity inversion of R11 George V, Cook, Ninnis, Mertz ice shelves.**

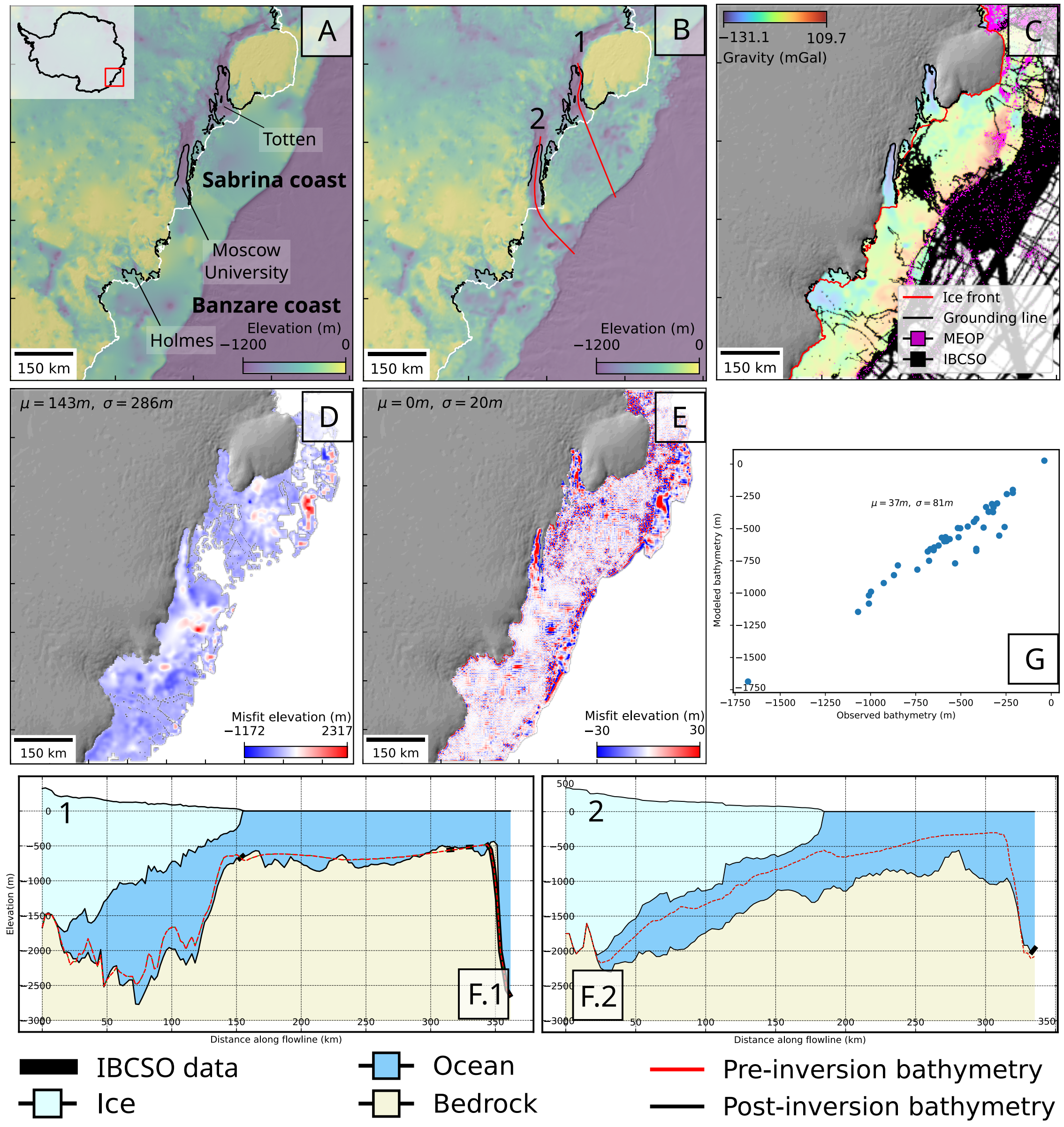

Figure S7l. **Gravity inversion method of R12 Sabrina, Totten and Moscow University Ice Shelf.**

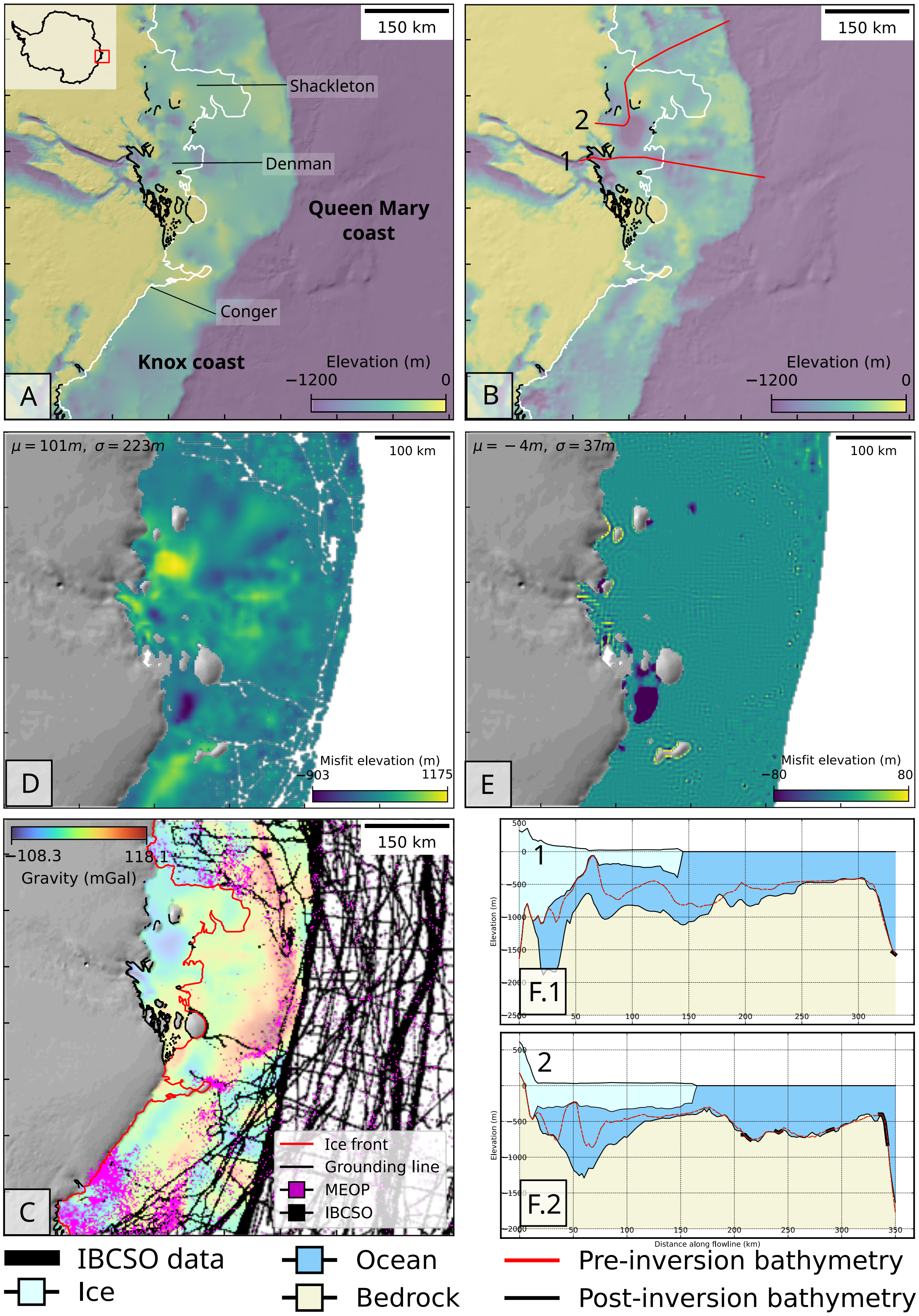

Figure S7m. Gravity inversion of R13 Queen Mary, Shackleton Ice Shelf.

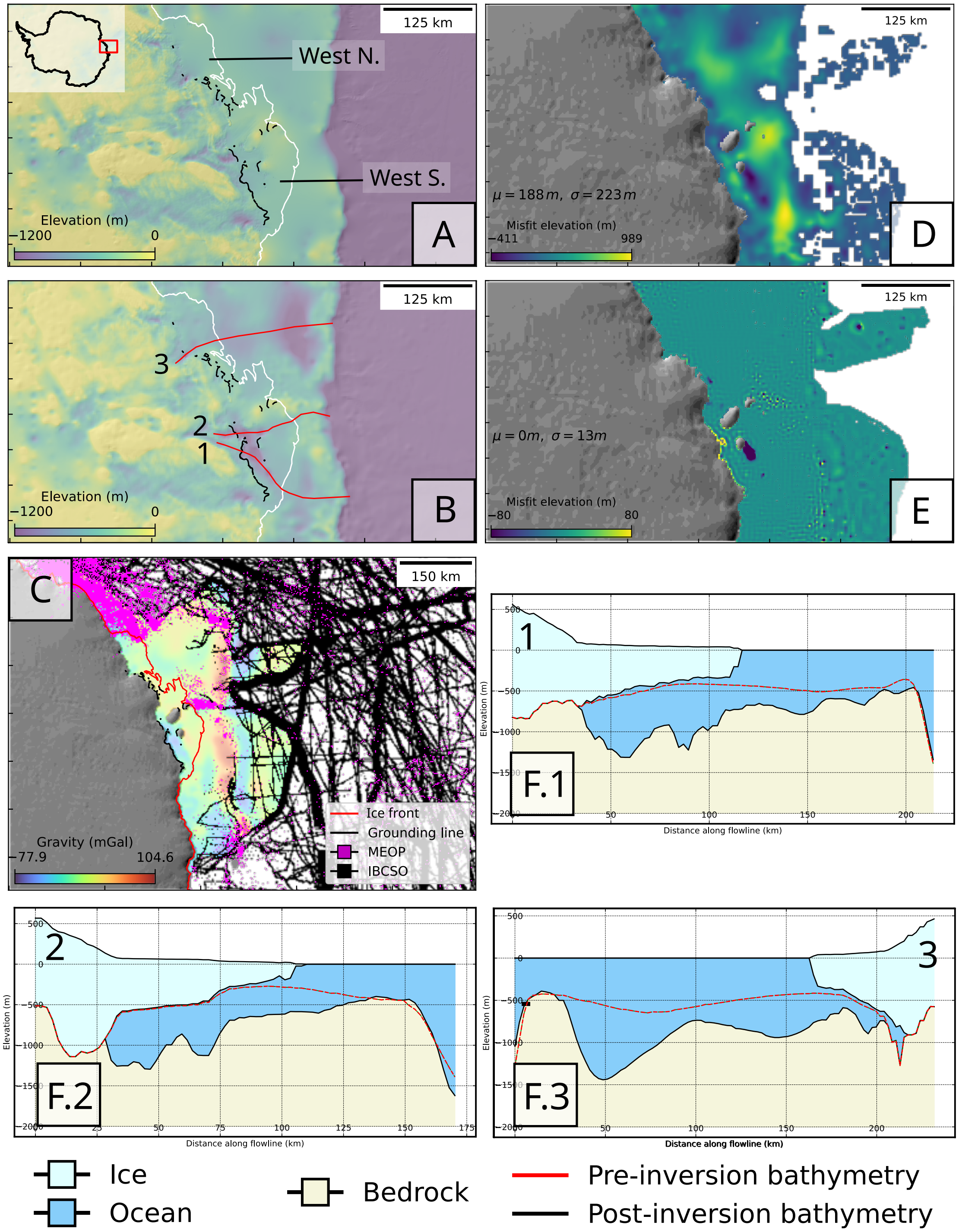

Figure S7n. **Gravity inversion of R14 Leopold, West Ice Shelf.**

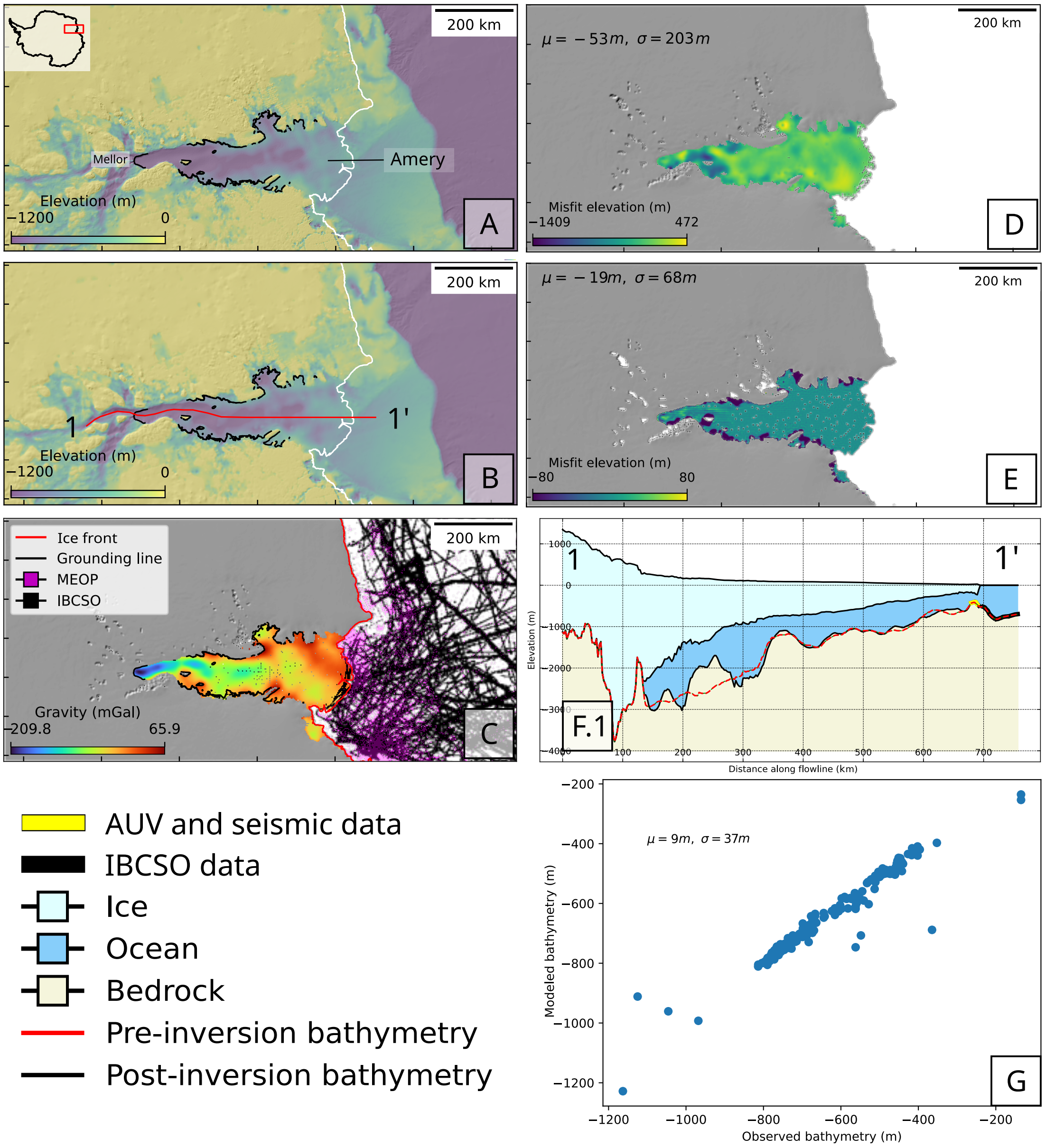

Figure S7o. Gravity inversion of R15 Amery, Amery Ice Shelf.

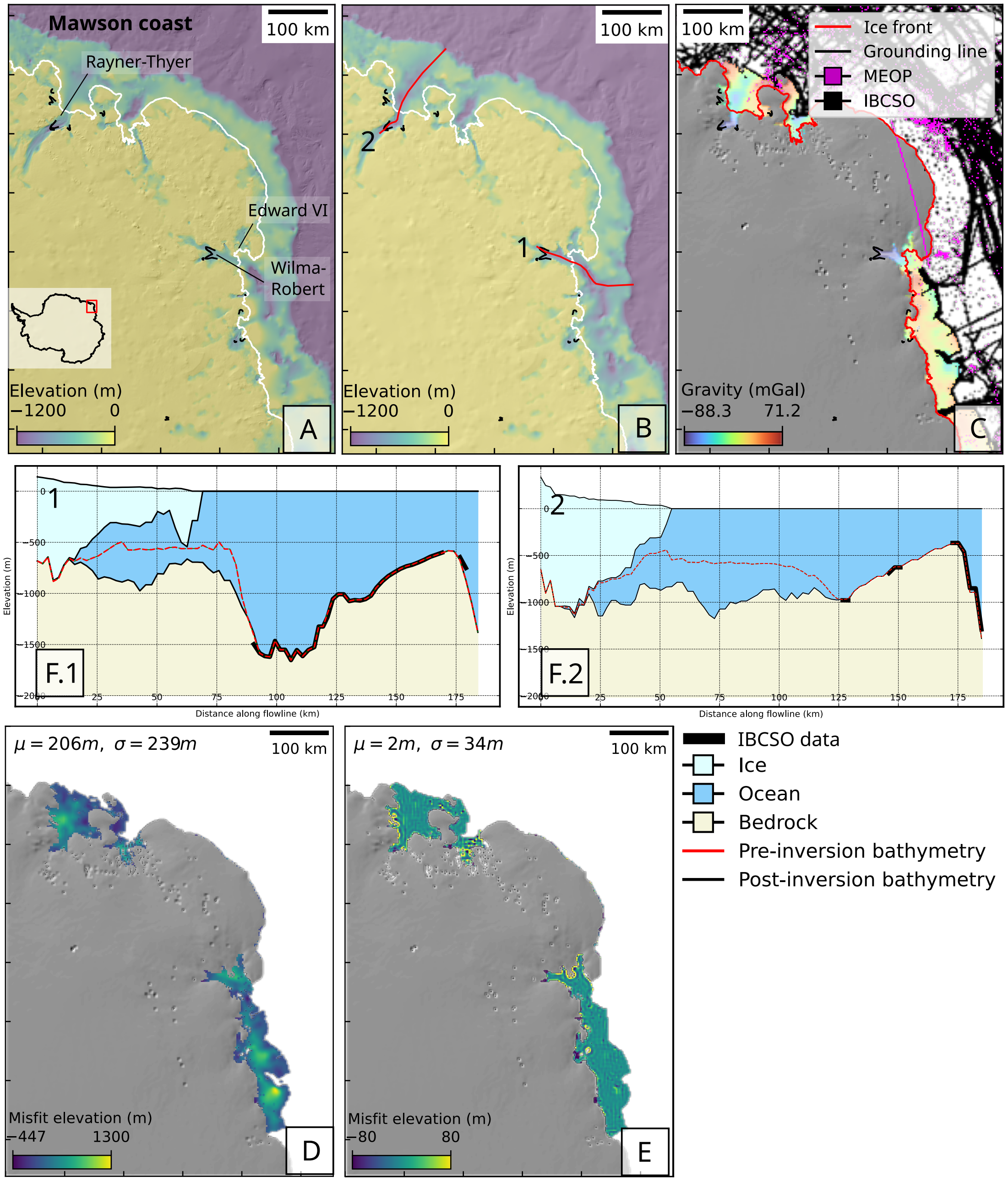

Figure S7p. Gravity inversion of R16 Mawson, Rayner-Thyer and Wilma-Robert-Downer ice shelves.

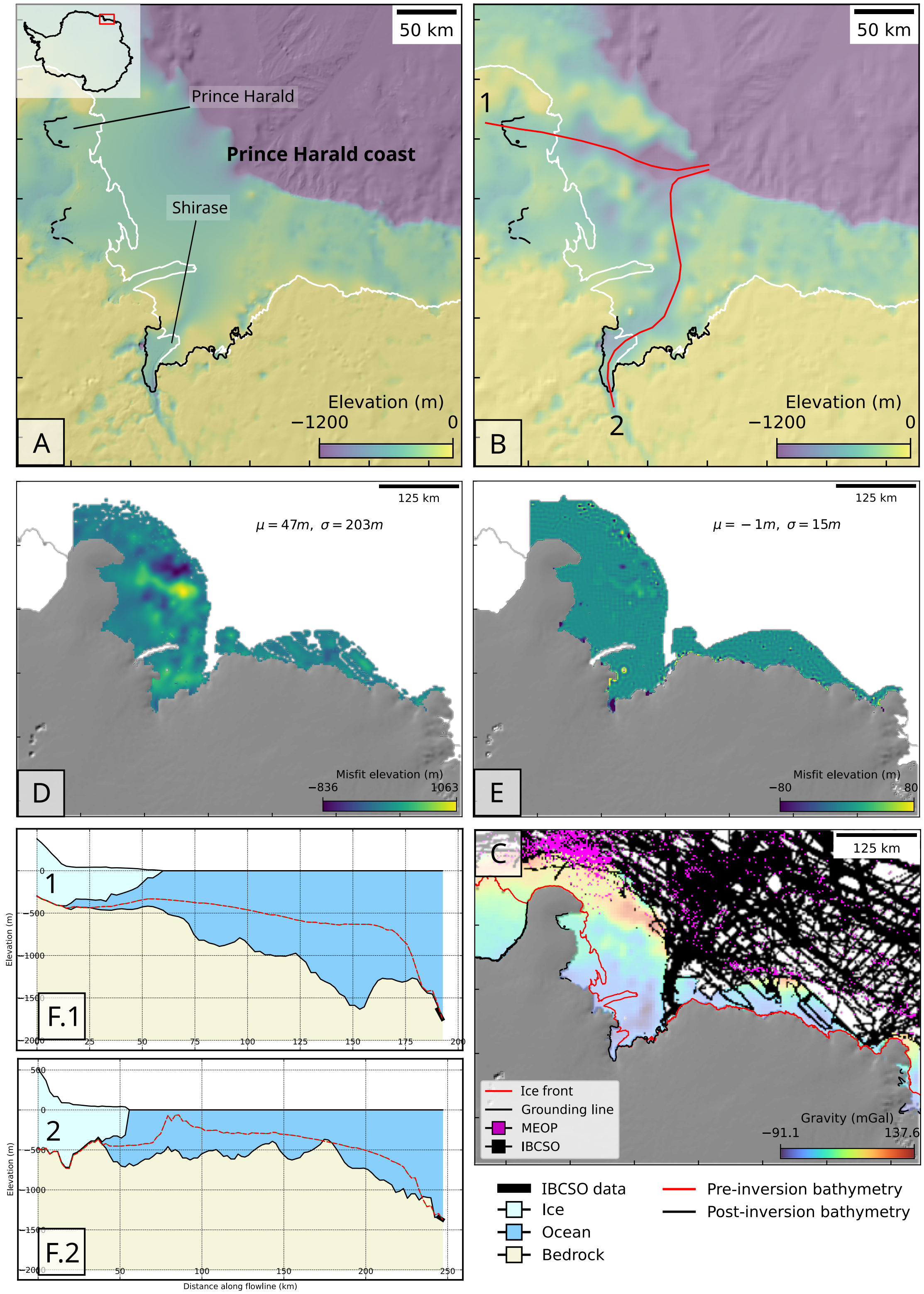

Figure S7q. **Gravity inversion of R17 Prince Harald, Prince Harald and Shirase ice shelves.**

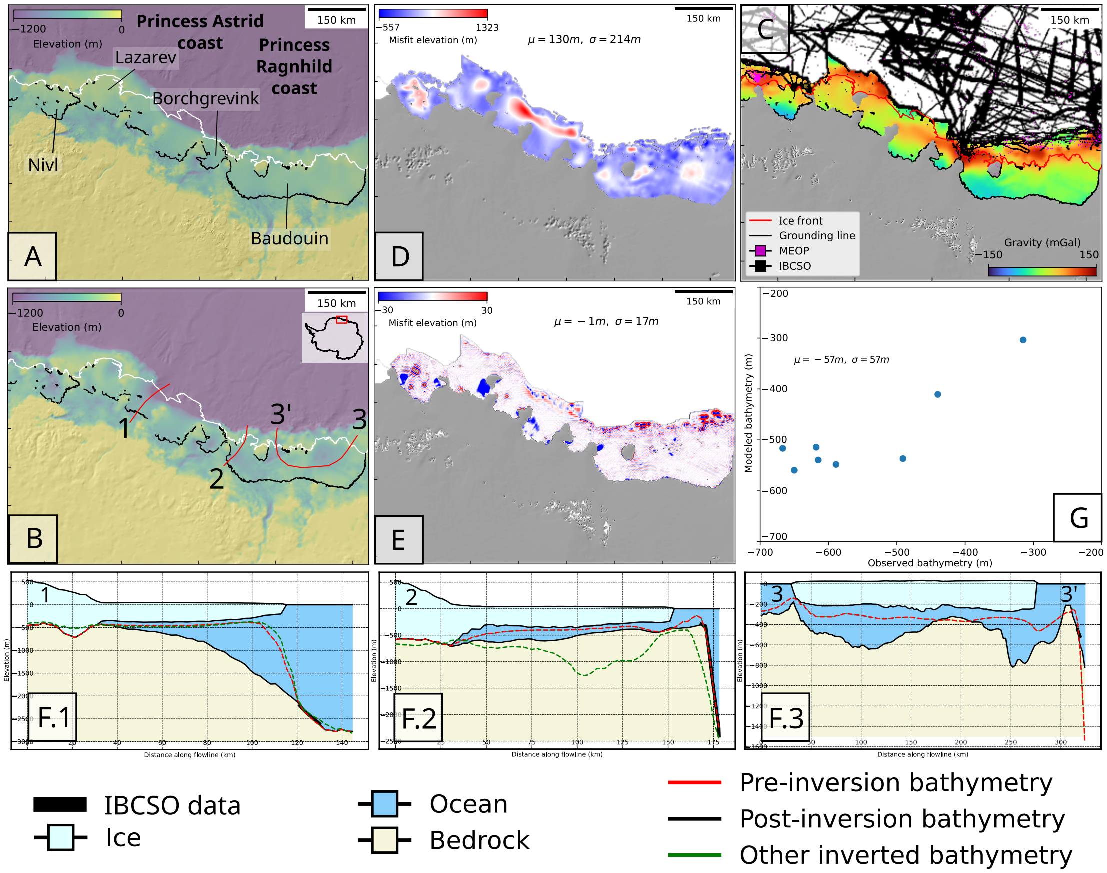

Figure S7r. **Gravity inversion of R18 Princess Ragnhild, Baudouin Ice Shelf.**

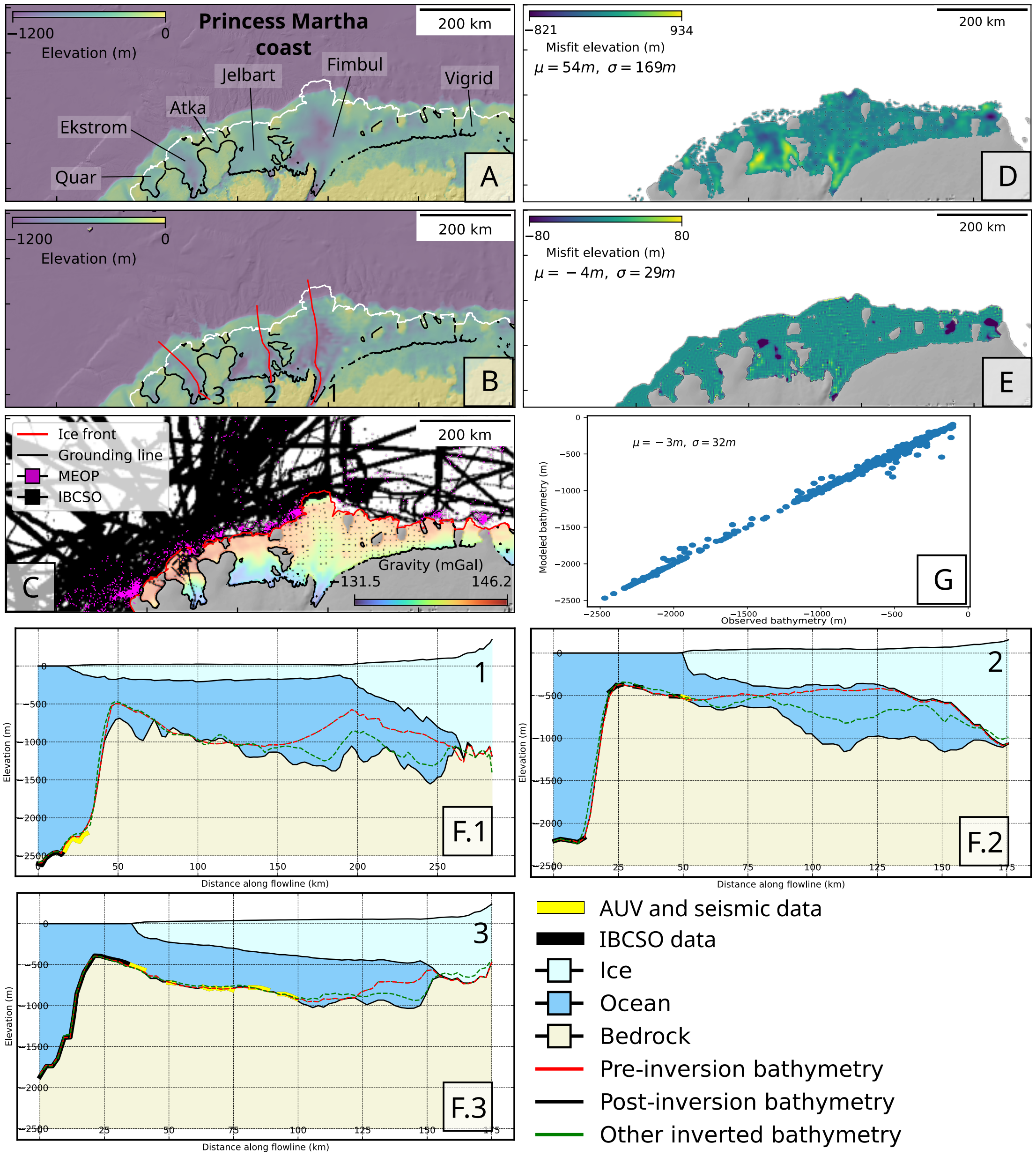

Figure S7s. **Gravity inversion of R19 Princess Martha, Fimbul Ice Shelf.**

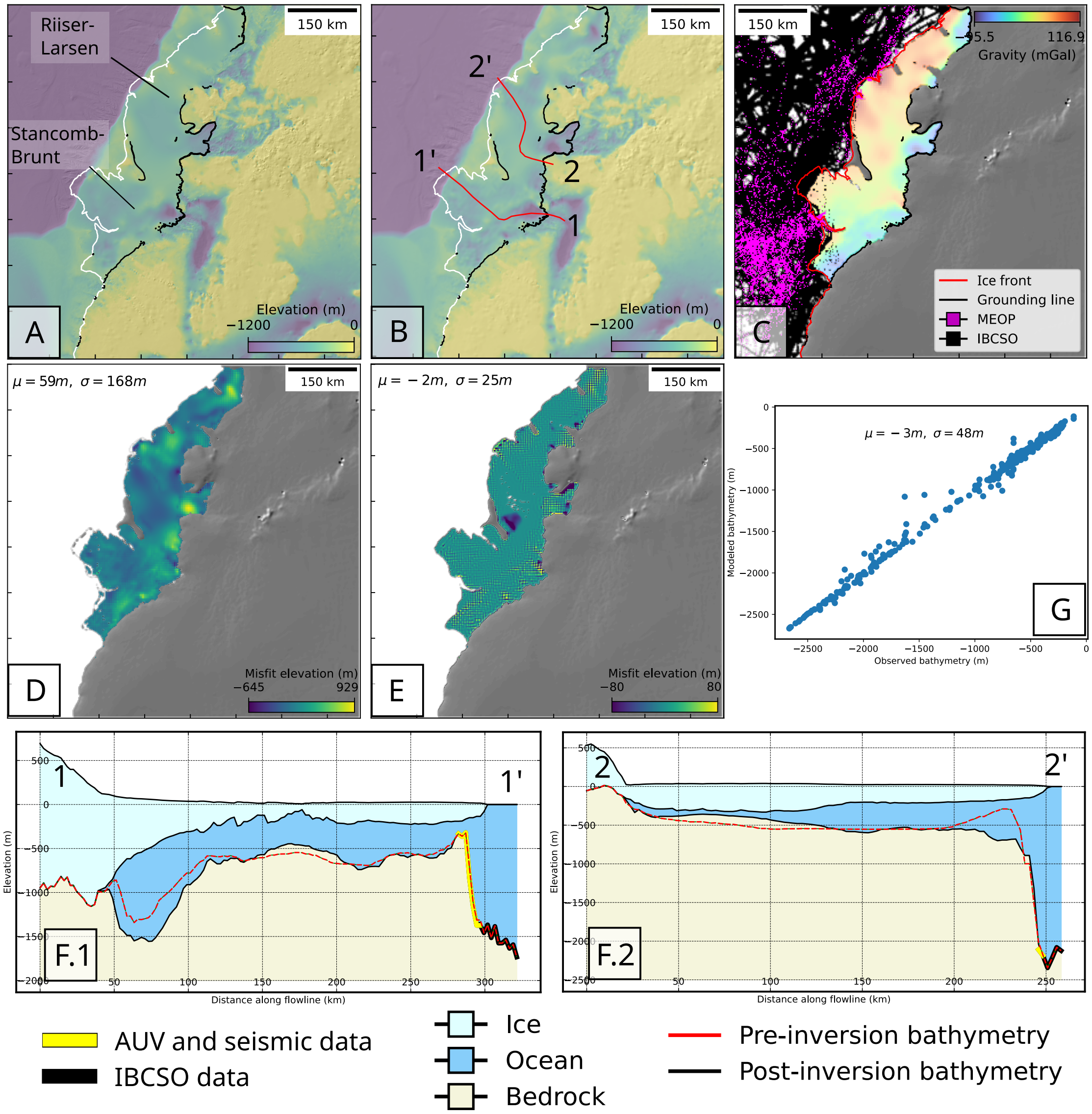

Figure S7t. **Gravity inversion R20 Brunt-Stancomb, Stancomb-Wills, Brunt, and Riiser-Larsen ice shelves.**

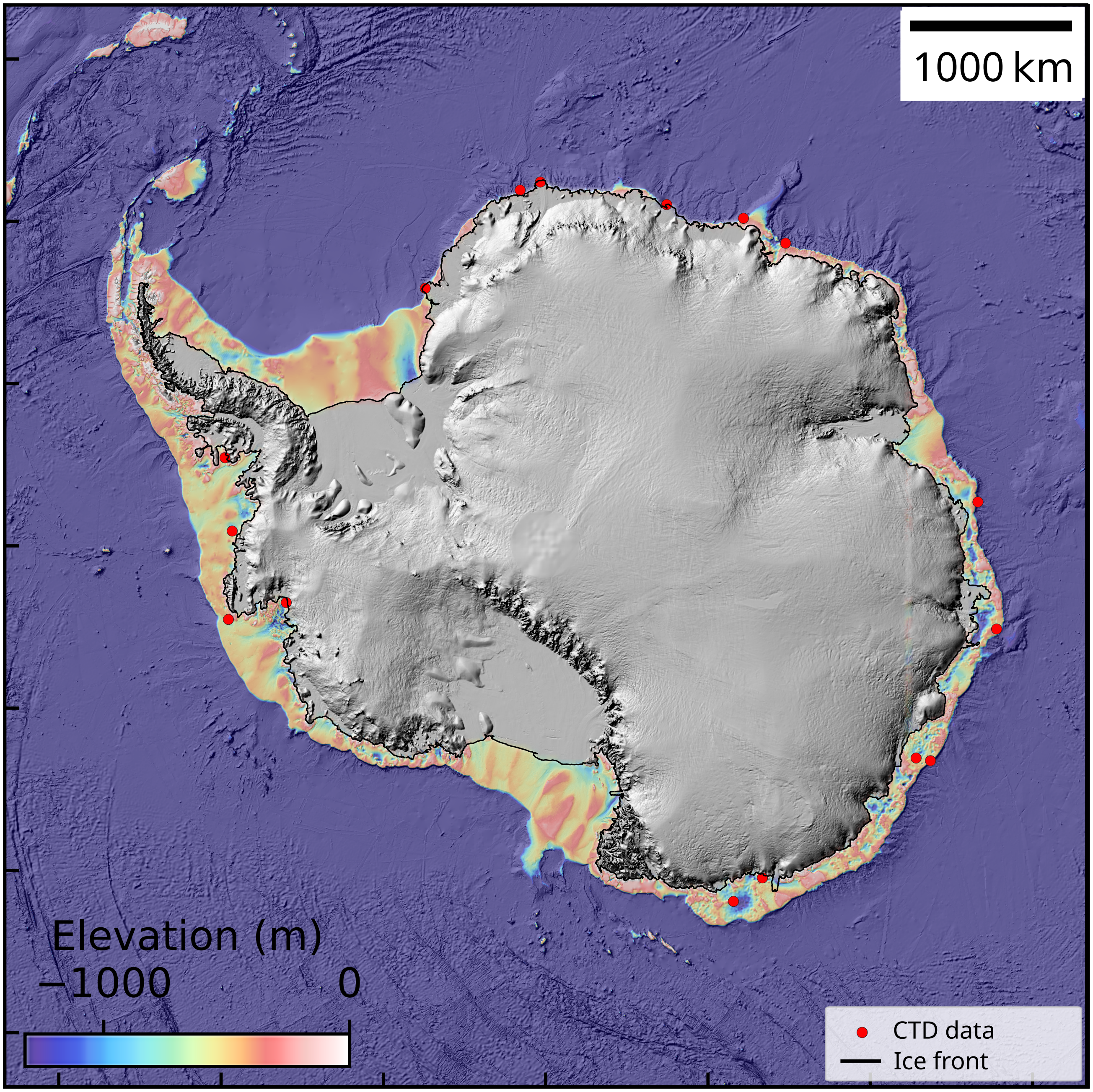

Figure S8. MEOP CTD data used in Figure 3. The seafloor is color coded from -1500 m (blue) to +1250 m (white) overlaid on the Antarctic surface in shaded relief. Ice shelf fronts are black. CTD locations are red dots.

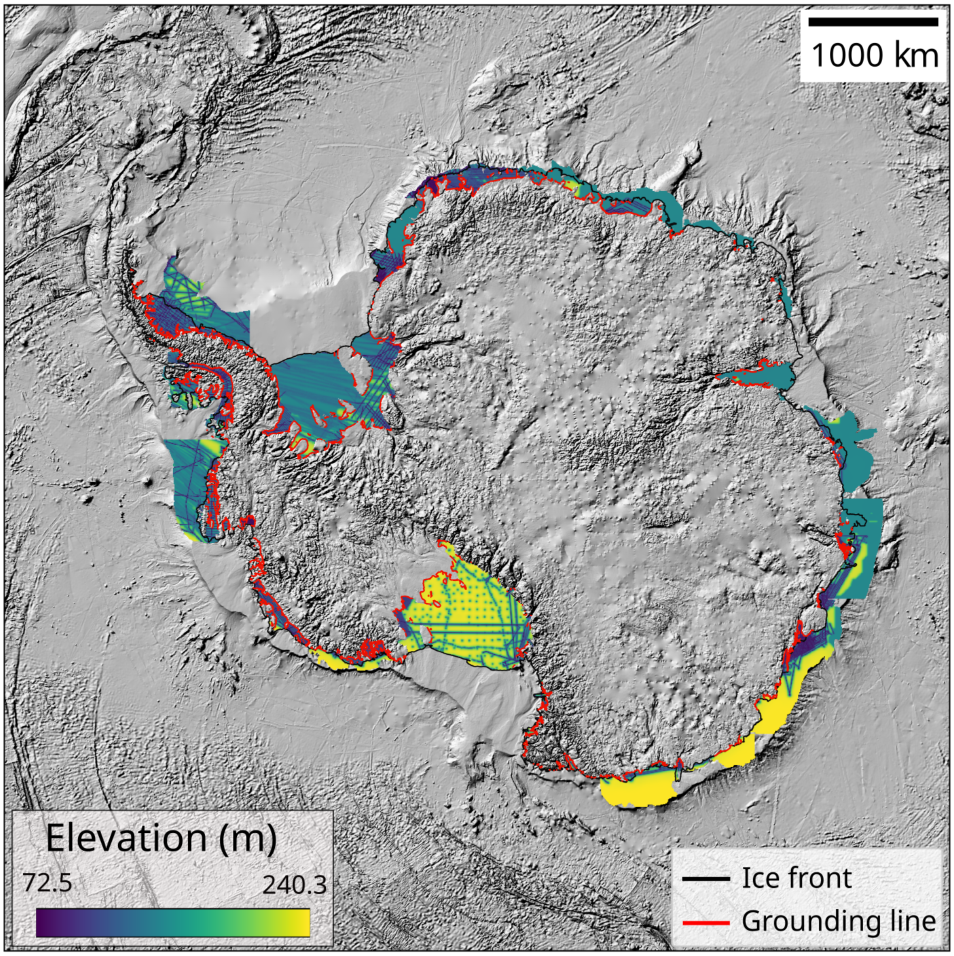

Figure S9. Estimate of the inversion elevation uncertainty map, as a function of the gravity standard deviation. The uncertainty is color coded from 72.5 m (blue) to 240.3 m (yellow).

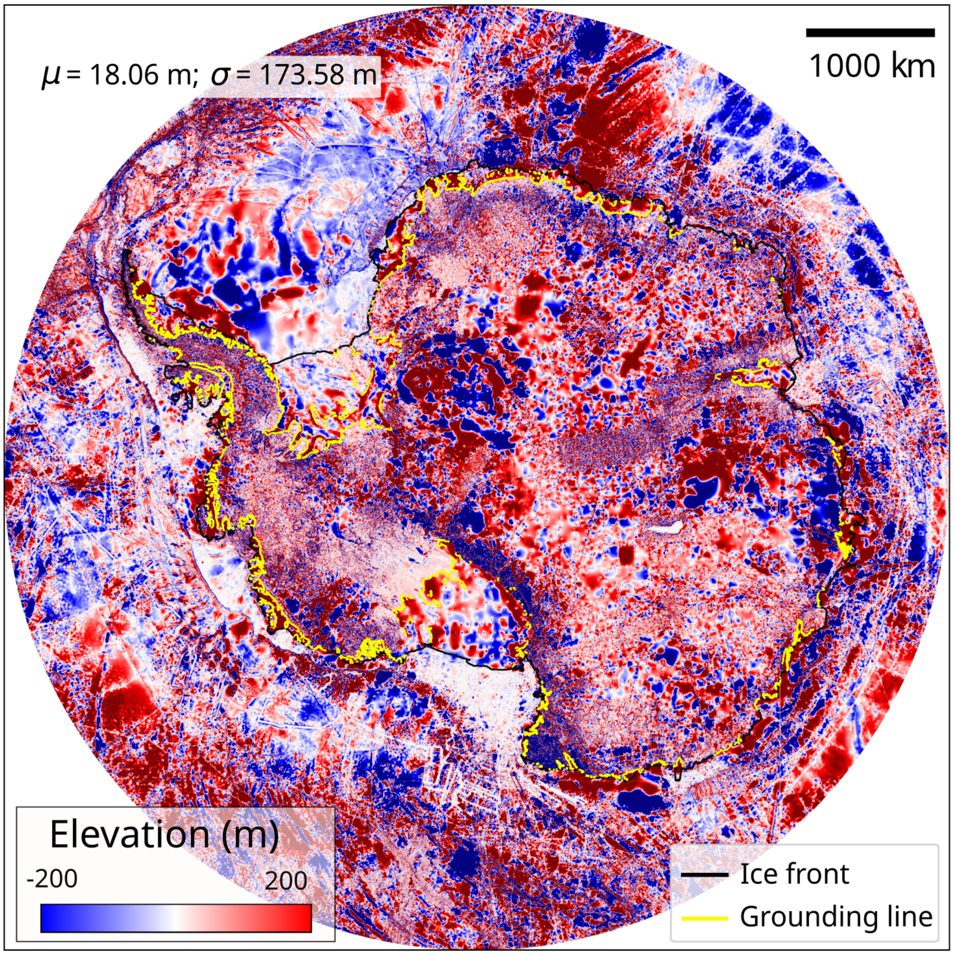

Figure S10. Difference in elevation between Bedmap2 and the ANTGG2021 bathymetry. The difference elevation is color coded from -200 m (blue) to 200 m (red). The mean difference of elevation and the standard deviation are shown.

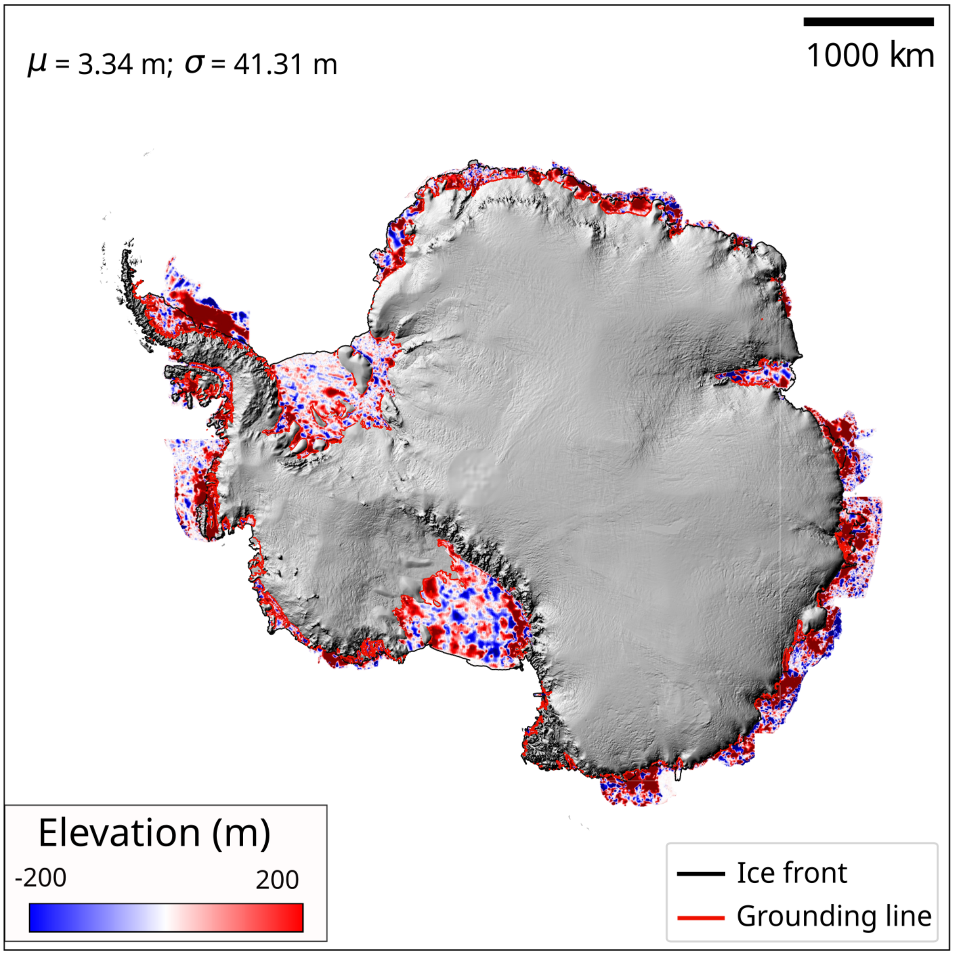

Figure S11. Difference in elevation between BedMachine and the ANTGG2021 bathymetry. The difference elevation is color coded from -200 m (blue) to 200 m (red). The mean difference of elevation and the standard deviation are shown.

References

1. Scheinert, M. *et al.* New Antarctic gravity anomaly grid for enhanced geodetic and geophysical studies in Antarctica. *Geophys. Res. Lett.* **43**, 600–610 (2016).

2. Sanso, F. & Sideris, M. (eds.) *Geoid determination - Theory and methods* (Springer Verlag Berlin Heidelberg, 2013).

3. Zingerle, P., Pail, R., Scheinert, M. & Schaller, T. Evaluation of terrestrial and airborne gravity data over Antarctica – a generic approach. *J. Geod. Sci.* **9**, 29–40 (2019).

4. Mayer-Gürr, T., Kvas, A., Klinger, B., Maier, A. & the GOCO team. The new combined satellite only model GOCO05s. *Geophys. Res. Abstr.* **17** (2015).

5. Hirt, C. & Rexer, M. Earth2014: 1 arc-min shape, topography, bedrock and ice-sheet models – Available as gridded data and degree-10,800 spherical harmonics. *Int. J. Appl. Earth Obs. Geoinf.* **39**, 103–112 (2015).

6. Moritz, H. (ed.) *Advanced physical geodesy* (Herbert Wichmann Verlag, Karlsruhe, 1980).

7. Zingerle, P., Pail, R., Willberg, M. & Scheinert, M. A partition-enhanced least-squares collocation approach (PE-LSC). *J. Geod.* **95**, 1–16 (2021).

8. Rosier, S. H. R. *et al.* A New Bathymetry for the Southeastern Filchner-Ronne Ice Shelf: Implications for Modern Oceanographic Processes and Glacial History. *J. Geophys. Res. Ocean.* **123**, 4610–4623 (2018).

9. Brisbourne, A. *et al.* An updated seabed bathymetry beneath Larsen C Ice Shelf, west Antarctic. preprint (2019).

10. Fretwell, P. *et al.* BEDMAP3 - Ice thickness, bed and surface elevation for Antarctica - standardised shapefiles and geopackages, DOI: 10.5285/A72A50C6-A829-4E12-9F9A-5A683A1ACC4A (2022).

11. Muto, A. *et al.* Subglacial bathymetry and sediment distribution beneath pine island glacier ice shelf modeled using aerogravity and in situ geophysical data: New results. *Earth Planet. Sci. Lett.* **433**, 63–75 (2016).

12. Jenkins, A. *et al.* Observations beneath Pine Island Glacier in West Antarctica and implications for its retreat. *Nat. Geosci.* **3**, 468–472 (2010).

13. Vaňková, I. *et al.* High Spatial Melt Rate Variability Near the Totten Glacier Grounding Zone Explained by New Bathymetry Inversion. *Geophys. Res. Lett.* **50**, e2023GL102960 (2023).

14. Galton-Fenzi, B., Maraldi, C., Coleman, R. & Hunter, J. The cavity under the Amery Ice Shelf, East Antarctica. *J. Glaciol.* **54**, 881–887, DOI: 10.3189/002214308787779898 (2008).

15. Eisermann, H., Eagles, G. & Jokat, W. Coastal bathymetry in central Dronning Maud Land controls ice shelf stability. *Sci. Rep.* **14**, 1367 (2024).

16. Eisermann, H., Eagles, G., Ruppel, A., Smith, E. C. & Jokat, W. Bathymetry Beneath Ice Shelves of Western Dronning Maud Land, East Antarctica, and Implications on Ice Shelf Stability. *Geophys. Res. Lett.* **47** (2020).
